# Unsupervised clustering based coronary artery segmentation

**DOI:** 10.1186/s13040-025-00435-y

**Published:** 2025-03-07

**Authors:** Belén Serrano-Antón, Manuel Insúa Villa, Santiago Pendón-Minguillón, Santiago Paramés-Estévez, Alberto Otero-Cacho, Diego López-Otero, Brais Díaz-Fernández, María Bastos-Fernández, José R. González-Juanatey, Alberto P. Muñuzuri

**Affiliations:** 1 FlowReserve Labs S.L., Santiago de Compostela, Galicia 15782 Spain; 2 CITMAga, Santiago de Compostela, Galicia 15782 Spain; 3 Group of Nonlinear Physics, University of Santiago de Compostela, Santiago de Compostela, Galicia 15782 Spain; 4 Cardiology and Intensive Cardiac Care Department, University Hospital of Santiago de Compostela, Santiago de Compostela, Galicia 15706 Spain; 5 Cardiology and Intensive Care Department, University Hospital of Pontevedra, Pontevedra, Galicia 36161 Spain; 6 Centro de Investigación Biomédica en Red de Enfermedades Cardiovasculares (CIBERCV), Madrid, 28029 Spain; 7 Instituto de Investigación Sanitaria de Santiago de Compostela (IDIS), Santiago de Compostela, Galicia 15706 Spain

## Abstract

**Background:**

The acquisition of 3D geometries of coronary arteries from computed tomography coronary angiography (CTCA) is crucial for clinicians, enabling visualization of lesions and supporting decision-making processes. Manual segmentation of coronary arteries is time-consuming and prone to errors. There is growing interest in automatic segmentation algorithms, particularly those based on neural networks, which require large datasets and significant computational resources for training. This paper proposes an automatic segmentation methodology based on clustering algorithms and a graph structure, which integrates data from both the clustering process and the original images.

**Results:**

The study compares two approaches: a 2.5D version using axial, sagittal, and coronal slices (3Axis), and a perpendicular version (Perp), which uses the cross-section of each vessel. The methodology was tested on two patient groups: a test set of 10 patients and an additional set of 22 patients with clinically diagnosed lesions. The 3Axis method achieved a Dice score of 0.88 in the test set and 0.83 in the lesion set, while the Perp method obtained Dice scores of 0.81 in the test set and 0.82 in the lesion set, decreasing to 0.79 and 0.80 in the lesion region, respectively. These results are competitive with current state-of-the-art methods.

**Conclusions:**

This clustering-based segmentation approach offers a robust framework that can be easily integrated into clinical workflows, improving both accuracy and efficiency in coronary artery analysis. Additionally, the ability to visualize clusters and graphs from any cross-section enhances the method’s explainability, providing clinicians with deeper insights into vascular structures. The study demonstrates the potential of clustering algorithms for improving segmentation performance in coronary artery imaging.

**Supplementary Information:**

The online version contains supplementary material available at 10.1186/s13040-025-00435-y.

## Background

Coronary artery diseases are a leading cause of mortality worldwide [1], underscoring the importance of advanced diagnostic methods [2]. Medical imaging, particularly computed tomography coronary angiography (CTCA), plays a pivotal role in non-invasively visualization of coronary arteries with the aid of contrast agents [3, 4]. This technique allows clinicians to assess and diagnose arterial lesions without the need for invasive procedures like catheterization and, at the same time, allows systematic scans over the whole affected population in order to search for underlying pathologies. However, the limitations of 2D visualization necessitate more detailed imaging; thus, generating a precise 3D geometry of the coronary arteries through CTCA segmentation is invaluable for clinical decision-making. Furthermore, these 3D geometries can be employed for fluid dynamics simulations, enabling the non-invasive calculation of essential parameters such as Fractional Flow Reserve (FFR) [2, 5, 6]. These simulations provide valuable insights that would otherwise require invasive procedures, thereby reducing patient risk and improving diagnostic accuracy.

Manual segmentation of these geometries is tedious and error-prone, prompting a surge in studies focusing on automatic coronary artery segmentation techniques in recent years. Currently, artificial intelligence (AI) has made significant inroads in the field of medical imaging. Specifically, there is a growing use of convolutional neural networks in 2D and 3D versions and supervised learning [7–13].

While this methodology is highly effective, it has several limitations. Firstly, it requires training with manually segmented images, which can be difficult to obtain, as it requires precise and expert annotation. Secondly, the training process demands specialized hardware, such as GPUs and substantial memory, to be efficient.

An alternative to these methods is to consider unsupervised learning techniques, such as region growing, snakes, level-set or thresholding algorithms.

For instance [14], presents a segmentation approach based on active contours, utilizing both local intensity information and the cumulative distribution function of input images. It emphasizes the issue of the kissing vessel artifact [15] and proposes an algorithm to correct it by tracking structures from previous slices. It achieves a Dice score of 0.776 on three patients but evaluates only the four main branches. Reference [16] employs cross-sectional images of the vessels, derived from centerlines calculated by its own algorithm, for segmentation using a levelset al.gorithm. Evaluated on the main branches of eight patients, it achieves a Dice score of 0.828. As a thresholding algorithm example [17], enables fully automatic segmentation using an iterative algorithm that calculates a global threshold for segmentation. The Dice score obtained on a dataset of 12 patients is 0.92.

In this work, we will focus on studies that have utilized clustering techniques for segmentation. By leveraging the inherent patterns in data without relying on pre-labeled images, clustering methods can effectively be used in medical imaging, addressing the limitations of supervised learning [18, 19].

Clustering algorithms are divided into partitioning and hierarchical algorithms. Partitional clustering groups data items into clusters based on an objective function, ensuring that items within the same cluster are more similar to each other than to those in other clusters. On the other hand, hierarchical clustering organizes data into a tree structure called a dendrogram. There are two types of hierarchical clustering algorithms: agglomerative and divisive. Agglomerative clustering follows a bottom-up approach, where individual data points are initially considered as separate clusters. These points are then progressively merged into larger clusters until a termination criterion is met or a single cluster containing all data items is formed [19].

Some studies use clustering algorithms for the segmentation of blood vessels in 2D. For instance [1, 20, 21, 22, 23], use clustering algorithms for the segmentation of vessels and other structures in retinal fundus images.

Focusing on CTCA images, [24] compares three methods for the segmentation and measurement of stenosis in 100 lesions from coronary angiographies: one based on thresholding, another based on edges and regions, and finally, the k-means clustering algorithm. After analysis, k-means is found to be the best-performing algorithm for evaluating lumen reduction. The segmentation and caliber measurement of vessels in coronary angiography is also studied by [25], who use the kmeans algorithm with k = 2 to separate the vessels from the background and obtain characteristic parameters of the vessel region, yielding good results in major vessels. Reference [26] employ a symmetrical radiation filter (SMF) and D-means clustering to reduce noise in the segmentation of coronary arteries. SMF, based on threshold gradient, is applied on axial, sagittal, and coronal planes to avoid relying on a single cross-sectional plane. A Dice coefficient of 0.9318 is obtained after evaluation on 210 CTCA volumes. Reference [27] use a multi-objective clustering algorithm to successfully segment and a toroidal model to track coronary arteries, with a Dice coefficient of 0.84 obtained after evaluation on 30 testing datasets from the Rotterdam Coronary Artery Algorithm Evaluation Framework [28]. Additionally, [29] use superpixel clustering to detect regions of interest and a graph cut segmentation approach to track and segment vessels. The coronary artery extraction is satisfactory only in vessels with high contrast and requires user input of seed points to continue the segmentation process.

In this paper, we propose an automatic and unsupervised segmentation method based on clustering techniques, specifically using the agglomerative Ward algorithm, and graph theory. In addition, this study includes a comparison of three methods for coronary artery segmentation. Two of these methods are based on clustering techniques, specifically in their 2.5D (3axis: axial, sagittal and coronal) and cross-section (perpendicular) versions. Additionally, we compare these clustering-based approaches with a neural network-based methodology, utilizing different architectures in their 2.5D and 3D modality.

By employing this unsupervised approach, we aim to effectively segment coronary arteries without the need for pre-labeled images or extensive manual intervention. The integration of clustering and graph-based methods allows for the identification and differentiation of distinct anatomical structures, enhancing the accuracy and efficiency of the segmentation process. This novel methodology offers a promising alternative to traditional supervised learning techniques, addressing their inherent limitations and paving the way for more advanced applications in medical imaging.

## Methods

In the present study, we propose to leverage the advantages of a two-dimensional (2D) clustering method to achieve three-dimensional (3D) segmentation of the coronary arteries. The algorithm effectively captures essential information from each input image (slice), including geometric characteristics, spatial positioning, and inter-cluster relationships within the image. This data is then represented as a graph, aiding in the identification and differentiation of structures within the image. The threshold for segmenting the vessel of interest is determined by selecting the corresponding cluster through an automated search within the graph. Finally, the combination of segmentation from all slices produces the 3D geometry, which is further post-processed to yield the final result.

The workflow proceeds through the subsequent steps:

1. Image extraction.
2. Application of Ward’s algorithm.
3. Graph representation and background removal.
4. Reapplication of Ward’s algorithm.
5. Threshold extraction.
6. Image segmentation.
7. Postprocessing.

### Image extraction

**Fig. 1:**
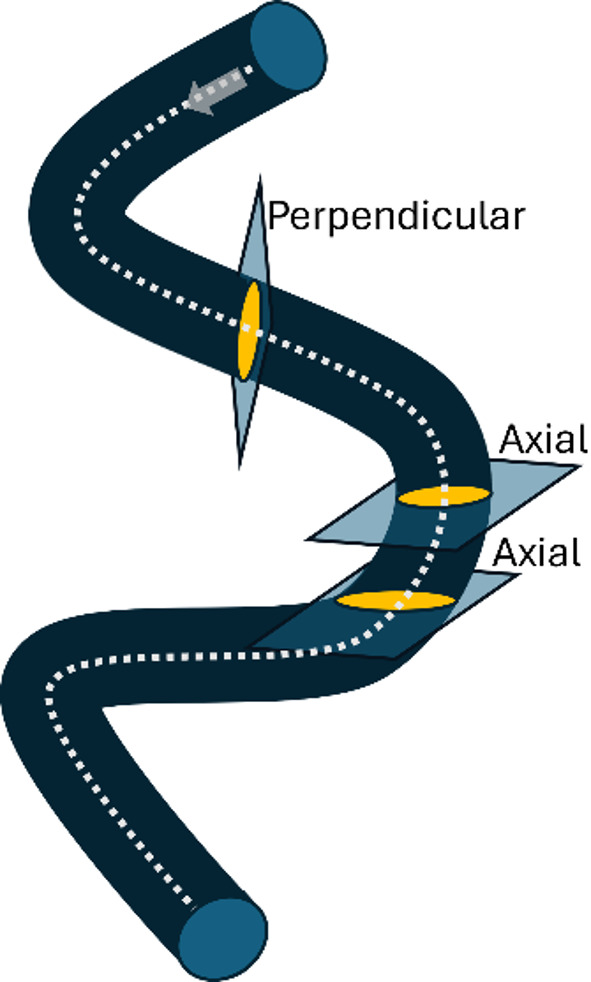
Diagram of a coronary vessel (dark blue). A dashed line depicts the centerline. Light blue indicates the perpendicular and axial planes, respectively. Yellow represents the intersection of the plane with the vessel. The grey arrow indicates the direction of blood flow

Vessel detection in CT coronary angiography (CTCA) based on only a single view (such as axial, for example) is a rather challenging task, as valuable information from neighbour slices is lost. Therefore, this study implements two distinct algorithms to address this limitation: 3Axis and Perp. The 3Axis algorithm processes the axial, sagittal, and coronal views of the vessel independently and then combines the information from each view to achieve a comprehensive analysis. On the other hand, the Perp algorithm uses cross-sectional (perpendicular) images of the vessel as its input, leveraging the localized information around the vessel lumen. These approaches ensure the integration of spatial and directional information to enhance vessel detection (see Fig. 1).

All images have a size of 32 × 32 pixels and are cantered on the vessel. This is achieved by extracting the centerlines of each vessel in a previous step. The extraction of centerlines is performed using the 3D Slicer software(version 5.2.2) with the Extract centerline module [30]. All the centerlines are resampled to 0.25 mm as maximal distance between nodes to ensure that no slice is lost in any of the 3 views. The initial, non-accurate segmentation for extracting the centerlines was performed semiautomatically by thresholding in the same software. It is noteworthy to mention that the use of centerlines facilitates the automation of the image extraction process. Additionally, the proposed algorithm is robust to small changes in the centerline position, meaning that slight variations in the extracted centerline do not affect the results significantly. This robustness allows the use of different centerline extraction methods without compromising the performance or accuracy of the algorithm. In the event of applying this method to the segmentation of other structures or images, centerlines may not be necessary.

### Dataset

The images have been provided by the Clinical University Hospital of Santiago de Compostela. Please refer to the Ethics Statement (Sect. Ethics statement) for details. The dataset used for result evaluation comprises 22 patients with diagnosed 30 lesions by clinicians (lesion set) and 10 patients without diagnosed lesions (test set) [11]. Details of the manual segmentation process are provided in Section S2 of the Supplementary Material. The number of images used for testing is 46,307 for the lesion set and 22,056 for the test set.

### Ethics statement

The study was conducted in full compliance with all relevant ethical guidelines and regulations. Detailed information regarding the Ethics Statement, including approval and consent processes, can be found in the Supplementary Material accompanying this article (Section S1).

### Application of Ward’s algorithm

The proposed method relies on Ward’s clustering algorithm [31], an agglomerative hierarchical clustering technique designed to minimize the total within-cluster variance. The algorithm begins by treating each data point as its own cluster and iteratively merges the two clusters that result in the smallest increase in within-cluster variance. This process continues until the desired number of clusters is achieved. Ward’s method ensures that clusters remain compact and well-separated, making it particularly effective for applications where precise boundaries between clusters are required.

While we initially expect two primary clusters—one for the vessel lumen and one for the background—the surrounding anatomical complexity necessitates a larger number of clusters. Coronary arteries are often bordered by various structures such as other vessels, myocardium, fat, and calcium, which must be accurately differentiated and excluded from the vessel of interest. Ward’s clustering excels in handling these nuances by leveraging its variance-minimization approach to form distinct and meaningful clusters.

Further details on the implementation of Ward’s method, as well as a discussion of why it was chosen over other clustering techniques, are included in the Supplementary Material (Section S3). This discussion also elaborates on the robustness and versatility of Ward’s method, making it particularly well-suited to the challenges presented in this application.

The aim of the study is to develop an algorithm that is as general as possible. Thus, we keep the same number of clusters for all patients. Selecting a fixed number of clusters is a challenging task that necessitates evaluating the algorithm across a diverse dataset. The goal is to distinguish different regions within the lumen, providing sufficient information to establish the cluster that will determine the vessel’s edge. Furthermore, to mitigate excessive variability in the image and thereby prevent the generation of new clusters in non-relevant structures, thus losing precision in the region of interest, all pixels in the input image with a Hounsfield Units (HU) value above (or below) a certain threshold are set to that threshold value. These thresholds are user-defined and are based on the HU values of the aorta. By default, these thresholds are set at 100 HU and 600 HU, respectively [32, 33, 34, 35].

### Graph representation and background removal

**Fig. 2:**
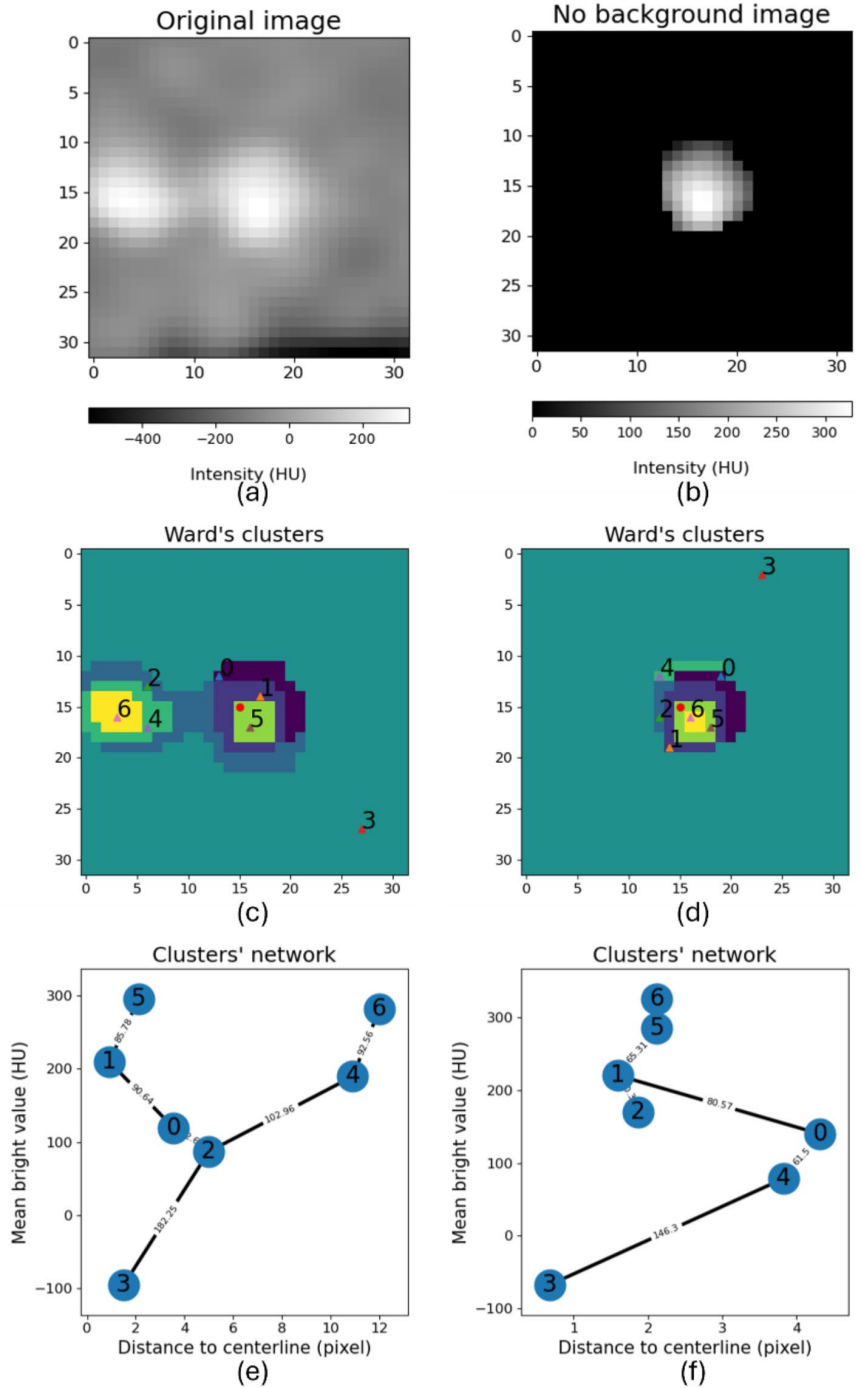
Background removal algorithm results. (**a**) Original perpendicular image of the vessel after a bifurcation. (**b**) Background removal algorithm result with input image (**a**). (**c**) Ward’s clustering extraction of input image (**a**). (**d**) Ward’s clustering extraction of input image (**b**). (**e**) Network extraction from clusters in (**c**). (**f**) Network extraction from clusters in (**d**). For both (**e**) and (**f**), the node number represents the cluster in (**c**) and (**d**), respectively. X-axis is the mean attenuation value of the cluster and y-axis is the distance in pixels between the centroid of the cluster and the centerline point. The connection weight between nodes represents the Euclidean distance between the corresponding clusters

A problem may arise if the vessel is close to other structures such us other vessels or the myocardium (also known as the kissing vessel artifact [15]. In that case, we lose cluster resolution due to the segmentation of these structures by the Ward’s method (see Fig. 2a and c). To address this issue, we implement a background removal algorithm. This involves identifying structures that do not belong to the vessel of interest and setting their Hounsfield value to the same as that of the background cluster. In this manner, the only variability in brightness resides within the vessel of interest. After this step we apply Ward’s clustering again to the cleaned image (see Fig. 2b and d).

The novelty of this background removal algorithm resides on the use of complex networks. The algorithm takes as inputs the original image (*img*), the clusters labels obtained by the Ward’s algorithm (label) with the same shape as the original image, the position of the image center in coordinates (*x*, *y*), named (*vesselCenter*), and the number of selected clusters (*nClusters*).

The information derived from all input parameters is integrated into a graph, where each node represents a cluster. Additionally, each node has two coordinates, facilitating its spatial representation on Cartesian axes. The x-coordinate signifies the distance from that centroid to the *vesselCenter* in pixels, while the y-coordinate represents the mean Hounsfield Units (HU) value of that cluster, computed in *img*. The connections between graph nodes are dictated by the connections between clusters. That is, if clusters are neighbors (have contiguous pixels), a connection will exist between them. Additionally, in Fig. 2e and f, weights can be observed on each of the edges between nodes; these represent the Euclidean distance between the two nodes. Once the graph is constructed, cycles are eliminated by calculating the minimum spanning tree using the Boruvka algorithm.

After generating the graph, our next step is to find the vessel path, containing the clusters of vessels in mean HU descending order from the centre to the edge of the vessel. To do that, we start by pinpointing the brightest cluster closest to the vessel centre, which likely represents the vessel interior or calcium deposits. From this starting point, we navigate through the graph, checking neighbouring nodes based on their mean HU values in descending order. We keep adding these nodes to a list until we hit nodes with an HU value below a set threshold, which is 100 by default. Consequently, clusters not belonging to the vessel of interest are those absent from this list. In the original image, pixels corresponding to the background are set to the same value, effectively masking the vessel (see Fig. 2b).

### Threshold and number of clusters selection

**Fig. 3:**
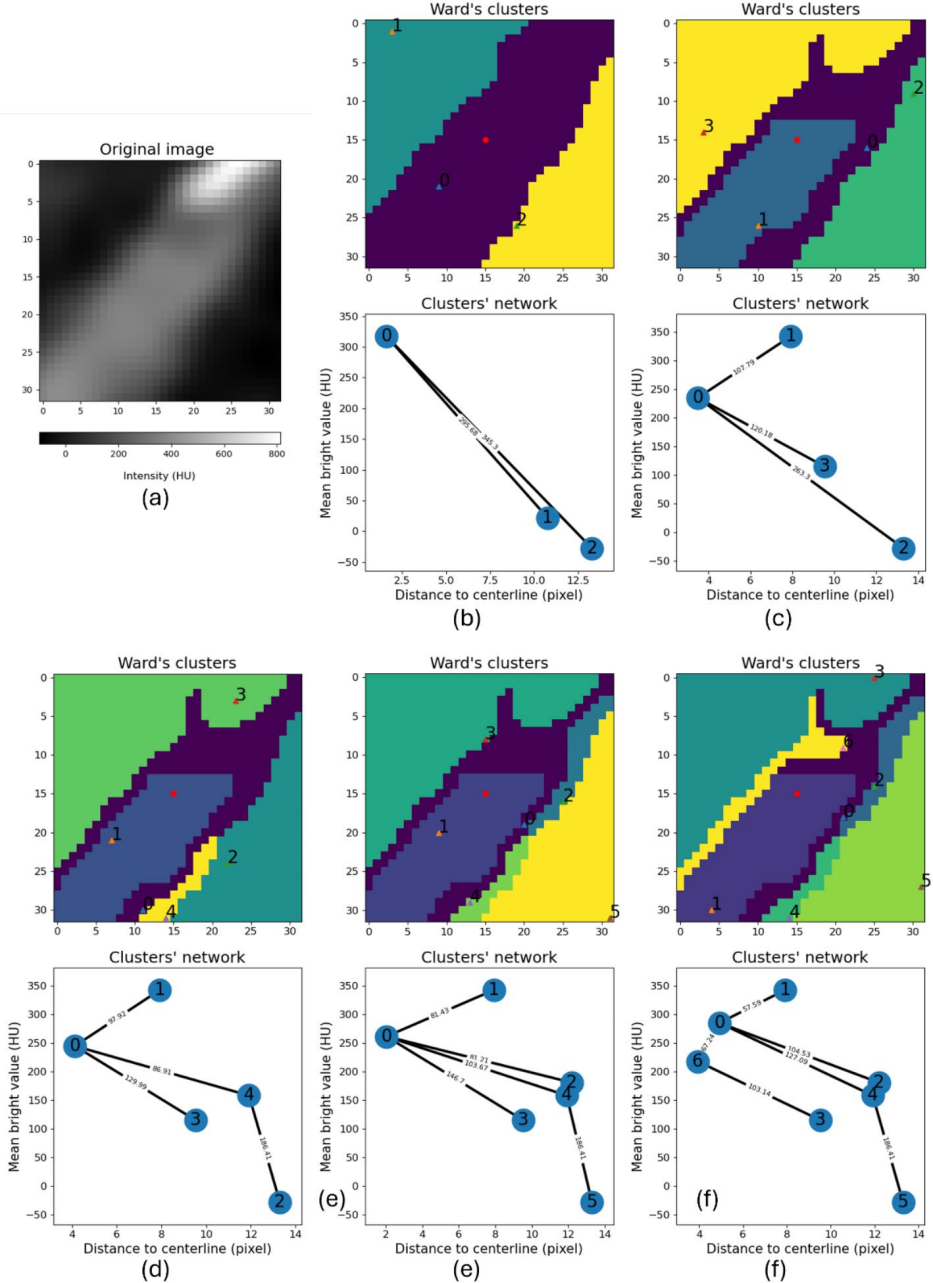
Results of the clusters obtained by the Ward algorithm in the second iteration (after removing the background of the image). (**a**) The original image and its attenuation values in Hounsfield Units (HU). Figures (**b**)-(**f**) show the clusters obtained by the Ward algorithm with different number of clusters (nC) at the top, and the graph associated with the clusters is shown at the bottom. X-axis is the mean attenuation value of the cluster and y-axis is the distance in pixels between the centroid of the cluster and the centerline point. The connection weight between nodes represents the Euclidean distance between the corresponding clusters. (**b**) nC = 3. (**c**) nC = 4. (**d**) nC = 5. (**e**) nC = 6. (**f**) nC = 7

Based on visual inspection by a medical image expert, the selected number of clusters was 7, this allows the differentiation of various regions within the main vessel and adjacent structures, permitting us to exclude them and focus solely on the vessel of interest. An example can be seen in Fig. 3. The selection of the number of clusters was determined by testing different values on vessel images from patients outside the study’s test and lesion datasets. This step was necessary to establish a parameter general enough to work across diverse cases while maintaining robustness.

When segmenting the coronary vessel, the threshold is determined from a sequence of brightness levels listed in the previous step (vessel path from Sect. Graph representation and background removal). This list is organized with the brightest clusters at the top, representing the interior of the vessel. As the list progresses, the clusters represent areas progressively closer to the edge of the vessel and eventually the background. Initially, these clusters are filtered by their brightness in Hounsfield Units (HU), retaining only those within the default range of [100, 600] HU. This range is critical because it includes the clusters associated with the coronary vessel, which is our target for segmentation. If the number of clusters within this range exceeds three, the third cluster is selected. If not, the last cluster in the list is selected. If there are no clusters within this specified brightness range, a default threshold of 150 HU is applied, allowing the user to check the segmentation.

### Image segmentation

Segmentation is performed based on the threshold calculated in the previous section for each point along the centerline. The reason for using a threshold value rather than the clusters themselves as a mask is that the result with the latter is irregular and does not follow the circular geometry of the vessel.

The methodology for segmenting coronary geometry from the selected threshold values varies depending on whether we employ the algorithm with perpendicular images or the one using the three planes (axial, sagittal, and coronal).

For the perpendicular method, segmentation is done with the threshold within a sphere of radius of 2 mm from the centerline point. In bifurcations, it is enforced to proceed only forward, that is, in the direction of the blood flow (see Fig. 1). Additionally, regions with lower threshold values are segmented first, ensuring that areas with higher thresholds (narrower) overwrite the others.

In the case of the algorithm with three views (axial, sagittal and coronal), segmentation is performed with a slice brush of radius 4 mm and a threshold in each plane. The reason for using a larger brush in this case is that it is not a spherical brush but a flat one that acts only on each view separately, thus avoiding overlap with other views. Additionally, the vessel view is no longer necessarily circular, as was the case in the perpendicular view, requiring a larger brush size to cover the surface.

Subsequently, all the planes are combined, and a *Median* smoothing of 1 mm is applied using the *smoothing* tool of *Segment Editor* module in 3D Slicer.

### Competing methods

In order to compare the capability of the clustering algorithm, we present some common neural network architectures used in medical image processing as could be VGG-19 [36], ResNet-50 [37], EfficientNet b2 [38] and U-Net++ [39]. The VGG-19, ResNet-50 and the EfficientNet b2 will be constructed as a classic U-Net model, where the encoder will be replaced by the encoder of the mentioned architectures. On the other side, the decoder used shall be equipped with usual upsampling and convolutional blocks to extract features from high complexity representations and also from the skip connections between encoder and decoder. U-Net + + implementation will be slightly different, because encoder and decoder used the same convolutional blocks and they were connected with a nested net of features maps between them. In addition, the encoder of all network has been pretrained with ImageNet dataset [40].

2.5D architectures [41] consist on take two or more 2D images with some spatial correlation and use it to predict volumetric complex structures, that are harder to recognize in 2D slices. To ensure that the models observe as much of the artery as possible for segmentation task, keeping computational complexity relatively low, we use as input dimension 3 slices (axial, sagittal and coronal) with shape 32 × 32 pixels.

In addition to the 2.5D models, we included three 3D segmentation networks to compare our method against established reference models. For this comparison, we selected two U-Net-based architectures and one Transformer-based model.

Following the approach described in [42], we implemented a simple 3D U-Net, incorporating convolutional blocks with two fused convolutional layers. Recent studies [43] have demonstrated that incorporating attention mechanisms in skip connections can enhance segmentation performance for certain tasks.

The U-Net-DR-LCT model [44] employs Local Contextual Transformers to capture critical features and utilizes Dense Residual blocks to preserve fine structural details. Finally, considering the increasing prominence of Transformer-based architectures in computer vision, we included the Swin-UNETR model [45] with features size embedding fixed at 24, a leading approach in medical image segmentation. The 3D models use blocks with shape 32 × 32 × 32 voxels as input.

A dataset of 32 patients manually annotated by a medical image expert (see Sect. Image extraction) was used. The dataset was divided into a training set and a test set, with detailed information provided as follow: The training set contains 27 patients $\:(\approx\:85\%$ of the whole dataset) while for the test set 5 patients ($\:\approx\:15\%$ of the whole dataset) were randomly selected. This dataset contains calcium plaque in the 5% of the cases, which implies a very unbalanced problem due to lack of plaque samples. Also, the lesion regions are a minimal part of the whole dataset, being less than 3% of the samples. The total number of centerline points used to extract image data is 52.4 K points.

Image resolution has been enhanced from an anisotropic volume of 0.62 mm to an isotropic volume of 0.25 mm through interpolation. This critical step enables the segmentation of small stenosed vessels with lumen diameters smaller than 0.60 mm.

To focus the view on the arteries, we center the three view representation input data on centerlines points. To mitagate the lack of critical zones as could be lesions and calcium plaque depositions, we perform augmentations on this type of images as could be rotations, flips, brightness changes and zooms, balancing the critical zones in the training set about to 25% of the initial samples. This results in a total of 65 K images between enlargements and originals. The only preprocess made was clipping values lower than − 200 HU and higher than 1150 HU, and then standarize data into [0,1] range using min-max normalization. This HU interval allows the model to learn some features of the background near the vessels and to recognize calcium to avoid segmentation in these areas. To check the performance with different HU ranges, 32 patients were completely split in training and validation sets, which implies 26 patients to train and 6 to validate the performance. All the models were trained with Tversky Loss [46], using $\:\alpha\:=0.4$ and $\:\beta\:=0.6$ to guarantee strong penalty for false positive and oversegmentation. The training loop iterates for 50 epochs, with an early stop after 15 epochs without improvement in validation loss. Adam was selected as optimizer such as an initial $\:lr=0.001$ with ReduceOnPlateau strategy to adapt the learning rate during the training process. Also, the augmentations were computed outside the training instances to secure fast data loading. All models were trained in 64 Intel Xeon Ice Lake 8352Y equipped with 1 NVIDIA A100 GPU. The specific encoders and their number of parameters are detailed in Table 1.

**Table 1:** Details of model architectures used. The number of total parameters and the trainable/non-trainable parameters are computed for each model implementation

Model	Nº of param. (M)	Trainable param. (M)	Non-trainable param. (M)
Efficient Net-b2	14.295	14.226	0.069
ResNet-50	51.605	51.505	0.099
VGG-19	29.062	29.058	0.004
U-Net++	9.163	9.163	0
3D U-Net	22.581	22.581	0
3D U-Net DR	10.700	10.700	0
3D Swin UNETR	15.703	15.703	0

The models are configured to output 2.5D images with shape 32 × 32 with probability values in [0,1] range. Due to the big number of points per patient, to reconstruct the coronary tree we store in parallel the number of times the model segments each voxel and the number of times the model sees the voxel. In addition, to take into account the proximity of the voxel segmented in each image, we multiply the stored values by a distance kernel before fitting them in the empty reference volume. This technique mitigates the unstability of the models when they predict far from the centre of the image and lets the 2.5D image closer to each voxel take more importance to segment. To consider a voxel coronary artery, the value obtanied has to be greater than 0.5.

### Results evaluation

The chosen parameters for result evaluation are based on the well-known true positive (TP), false positive (FP), true negative (TN), and false negative (FN). From these, the following metrics are derived:

- Dice$$\:=\frac{2\times\:TP}{2\times\:TP+FP+FN}$$
- IoU $$\:=\frac{TP}{TP+FP+FN}$$
- Precision $$\:=\frac{TP}{TP+FP}$$
- Recall $$\:=\frac{TP}{TP+FN}$$

All coefficients range between 0 and 1. The Dice coefficient measures the similarity between the predicted and true segmentations, where 1 indicates the perfect match. The intersection over union (IoU) coefficient measures the ratio of the intersection area to the union area between the predicted and true segmentations, where 1 indicates perfect overlap and 0 no overlap. Additionally, precision measures the accuracy of positive predictions. Is the ratio of true positives to the total predicted positives. It reflects the ability of the model to avoid false positives. Finally, recall measures the ability of the model to capture all positive instances. Reflects the ability of the model to recognize the vessel.

## Results

In this section, we present the results of our study, beginning with a comparison between the number of clusters used to run Ward’s algorithm (Sect. Selection of the number of clusters). This is followed by a performance comparison between the clustering methods (3Axis and perpendicular (Perp)) and the neural networks employed in our analysis (Sect. Test set). The explainability and clinical significance are in Sect. Explainability and Clinical Value of the Proposed Method. The computation time and efficiency analysis are in Sect. Efficiency Analysis. In addition, given the significance of the lesion region in coronary artery analysis, our evaluation also focuses on the lesion set and specifically examines outcomes within the lesion region (Sect. Lesion set).

### Selection of the number of clusters

The Ward algorithm, like other clustering algorithms, requires the final number of clusters as an input parameter. As discussed in Sect. Application of Ward’s algorithm, our goal is to develop a general algorithm, and therefore, we aim to maintain a fixed number of clusters. This presents a challenge, as each image and patient have unique characteristics in terms of shape, brightness, geometry, lesions, and more.

To determine the appropriate number of clusters for the algorithm, multiple tests and visual inspections were conducted by an expert on various samples from the dataset. An example of this process is shown in Fig. 3. The original image shows a section of the vessel containing calcium. With 3 clusters (nC = 3), we can distinguish the two regions of the background and the vessel (including the calcium). With nC = 4, we successfully eliminate the calcium, making it part of the background thanks to the background removal algorithm. From this point, our goal is to achieve greater precision at the vessel’s edge, which is determined when nC = 7.

### Test set

**Fig. 4:**
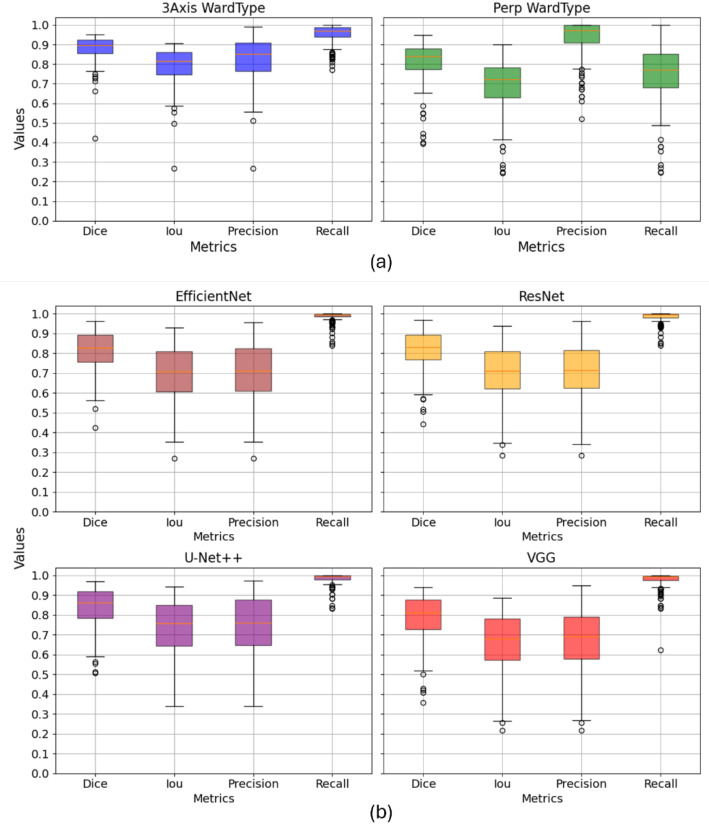
Box plot displaying the mean parameter values, Dice, IoU, Precision and Recall, obtained by the segmentation algorithms in the test set. (**a**) Clustering segmentation algorithms in three views (axial, sagittal, and coronal), referred to as 3Axis, and the segmentation algorithm in the perpendicular view, referred to as Perp. (**b**) Neural network architectures, EfficientNet, ResNet, U-Net + + and VGG

Figure 4 presents a comparison of the performance between the clustering algorithms and the neural networks investigated in this study, showing box plots where the values of the metrics computed at each centerline point are evaluated.

Our focus lies on the analysis of precision and recall metrics. It’s important to note that recall measures how much vessel we have been able to recognize, while precision indicates how much of that recognized vessel is accurate. High recall values and low precision values may suggest oversegmented vessels, meaning they are wider than they should be. Conversely, low recall values and high precision values may indicate undersegmented vessels, meaning they are thinner than they should be. These metrics provide valuable insights into the segmentation performance and aid in identifying potential issues such as over- or undersegmentation in the vessel structures.

Figure 4a illustrates the results obtained from the clustering methods 3Axis and Perp. A noticeable distinction arises in terms of recall and precision metrics. Specifically, the recall value is up to 20% higher (mean value: 0.95) in 3Axis compared to Perp, while the precision value is 10% lower (mean value: 0.83). This suggests that 3Axis demonstrates a greater ability to identify the majority of the vessel but also exhibits a higher number of false positives. Consequently, in this scenario, the vessel tends to be oversegmented. The Dice coefficient, which places more emphasis on true positives (TP), exhibits a notable difference of 10% compared to the Intersection over Union (IoU) metric. Notably, the 3Axis clustering method demonstrates superior performance in both Dice coefficient (mean value: 0.88) and IoU (mean value: 0.79) compared to the Perp method (0.81 and 0.7, respectively). This discrepancy can be attributed to the fact that Dice coefficient prioritizes the agreement between predicted and ground truth segmentations, resulting in higher scores for methods that excel in accurately capturing true positives.

Now we compare the performance of the Ward method and the 2.5D neural networks. One notable observation is the high recall achieved by the neural networks, indicating their ability to recognize almost all vessels. However, the precision of the neural networks is lower compared to that obtained with the Ward method, with the mean value being reduced by over 10%. This suggests that the neural networks tend to oversegment the vessels. Additionally, fewer outliers (scatter points in the figure) are observed with the Ward method, and the neural networks exhibit longer whiskers in the box plots, indicating greater variability in their results.

The Ward clustering method demonstrates strong performance, comparable to advanced deep learning approaches like the 3D U-Net and surpassing the 3D U-Net DR in accuracy. The 3D U-Net, which incorporates contextual information from neighbouring slices, achieves a mean Dice coefficient of 0.88 (std: 0.0888), closely matching the results of the 3Axis method. In contrast, the 3D U-Net DR delivers a lower performance, with a mean Dice coefficient of 0.6 (std: 0.23), indicating its limitations in this context.

**Fig. 5:**
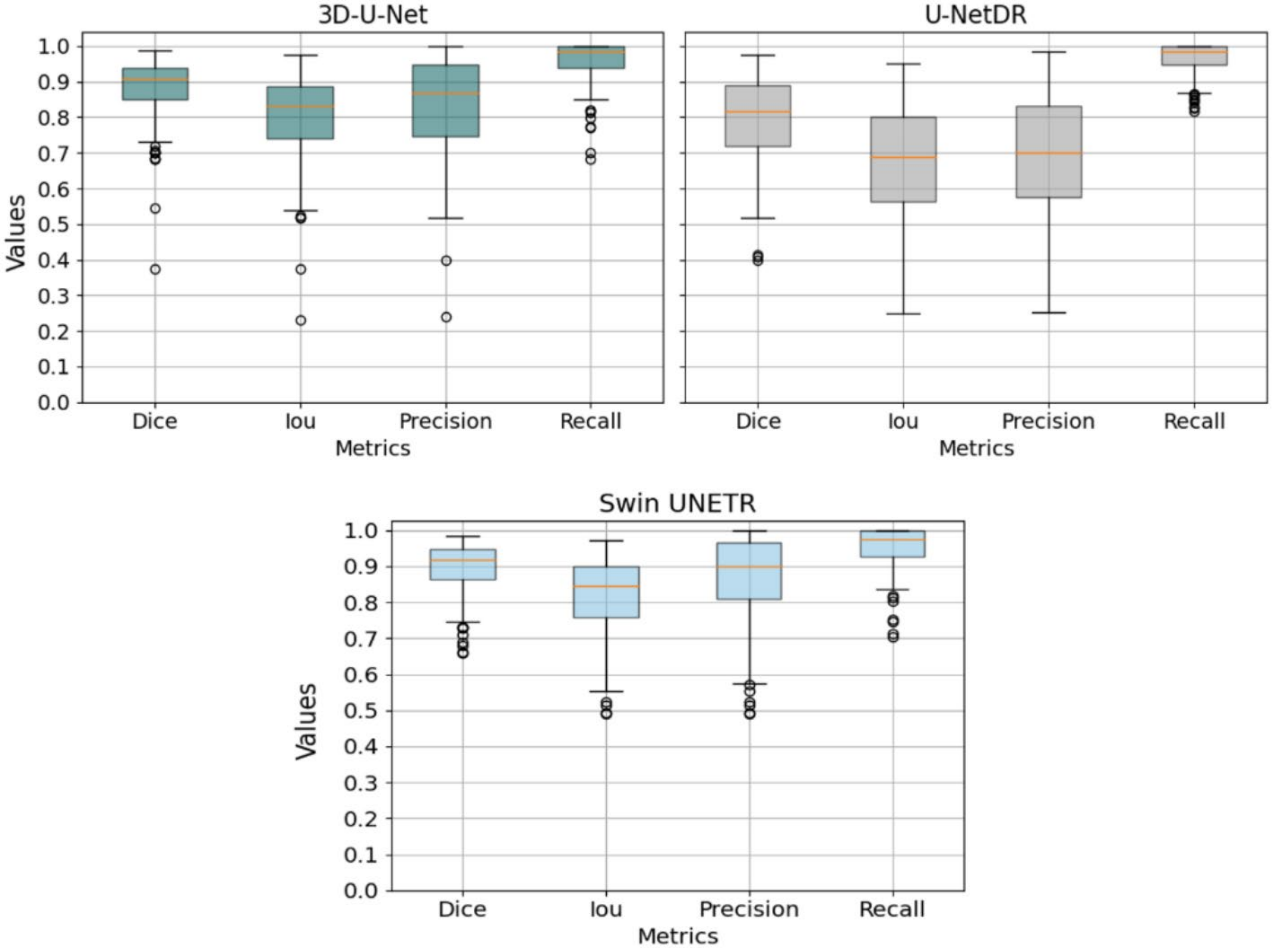
Box plot displaying the mean parameter values, Dice, IoU, Precision and Recall, obtained by the segmentation algorithms in the test set by the 3D neural network models: 3D U-Net, 3D U-Net DR and Swin UNERT

Notably, the Swin UNETR is the only model to exceed the 3Axis method’s performance, with a mean Dice coefficient of 0.8978 (std: 0.0706). However, this small improvement comes with significantly higher computational complexity due to the transformer-based architecture. This comparison highlights the balance achieved by the Ward clustering method, offering robust segmentation results with a more straightforward and efficient approach (see Fig. 5).

Hausdorff Distance and Mean Surface Distance (MSD) metrics can be found in Supplementary Material, Section S6.

**Fig. 6:**
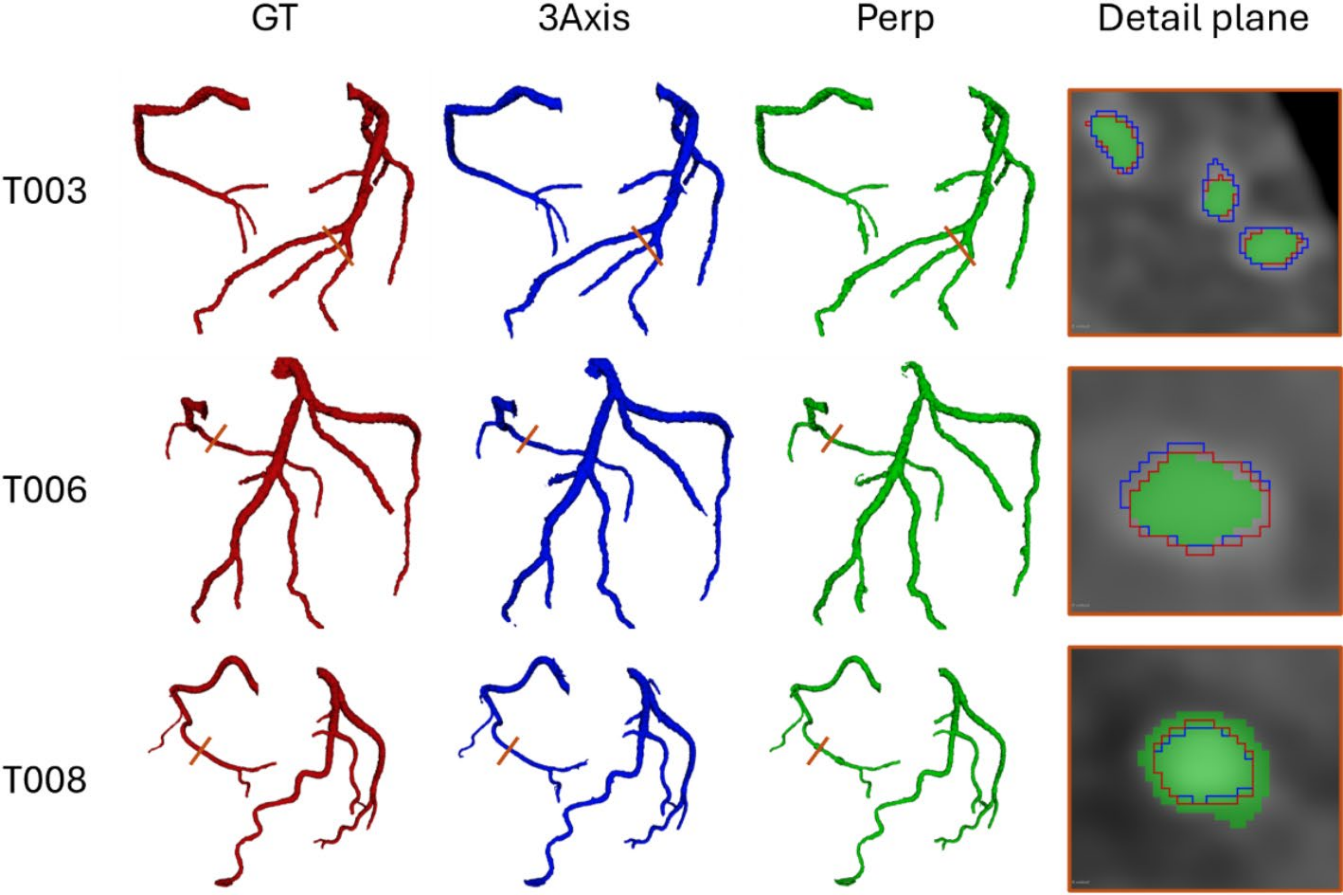
Results of the prediction for test patients T003, T006, and T008 (in rows). The columns represent the ground truth (red), segmentation using the 3Axis clustering method (blue), segmentation using the perpendicular clustering method (green), and the detail plane, depicted with an orange line in the previous geometries, in the 2D images

For a comprehensive understanding of these findings, Fig. 6 presents a 3D visualization depicting the ground truth geometries (red) alongside the corresponding predictions generated by the 3Axis (blue) and Perp (green) algorithms. Within the detailed plane, it is evident that the 3Axis algorithm tends to oversegment vessels in rows 1 and 2. Conversely, row 3 displays a region where the Perp algorithm demonstrates oversegmentation. These instances occur even in the absence of a lesion, indicating a potential limitation in algorithm robustness. This phenomenon may arise due to the computation of the perpendicular plane, which relies on the smoothness of the centerline. It could introduce small variations in the threshold determination. Additionally, patient-specific parameter settings may require adjustment, as they can influence the segmentation accuracy. Both factors suggest that fine-tuning and individualized calibration can improve performance.

### Explainability and clinical value of the proposed method

**Fig. 7:**
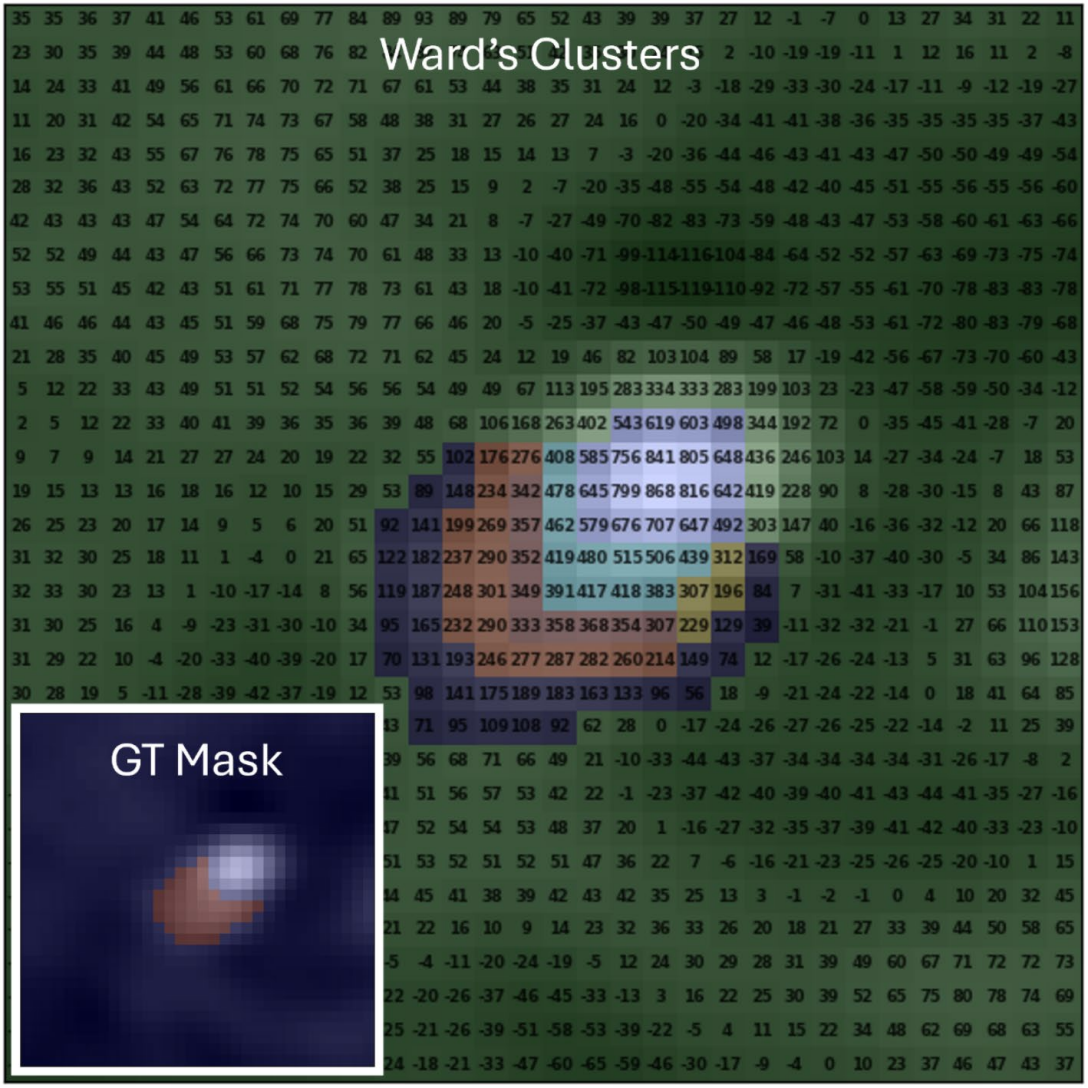
Results of the clusters obtained using the Ward algorithm after applying background removal and the corresponding ground truth mask, for a vessel affected by a lesion due to a calcium plaque in axial view. The brightness value of each pixel in the original image is displayed as its corresponding HU value directly on the pixel

The clustering method proposed in this study offers a high degree of explainability, making it a valuable tool for clinicians in understanding and interpreting medical images. The use of the Ward clustering algorithm allows for clear identification and segmentation of different regions in the image based on the Hounsfield Units (HU) values, which are directly related to tissue composition and density. In the example provided in Fig. 7, the vessel with a lesion due to a calcium plaque is displayed, with each pixel showing its corresponding HU value. This direct mapping of HU values to tissue characteristics allows for a transparent understanding of the algorithm’s decision-making process.

The clustering method not only effectively identifies the background (green cluster) but also highlights the calcium deposit (violet cluster), demonstrating its ability to detect specific pathological features such as lesions. The order in which the clusters are interpreted is determined by the graph, which accurately arranges them from the vessel’s interior to its edge (see Figure S4 in Supplementary Material). This structured approach enhances the method’s ability to delineate regions based on decreasing brightness (HU values), further aiding in uncovering additional areas of interest. These areas, often indicative of potential lesions or other abnormalities, can serve as early warnings to medical professionals, providing valuable diagnostic insights. More examples can also be found in Section S4 of the Supplementary Material.

### Efficiency analysis

In terms of speed, once the centerlines are extracted, the neural networks take around 7 min to segment 2.5D patches and reconstruct it again, while Ward algortihm need around 20 min to complete the task, which is rather slow than the neural network. Both times where measured when running the code in 3D Slicer v5.2.2 until obtaining the segment visualization.

On the other hand, the 3D U-Net, a 3D-based method, predicts the segmentation in approximately 3 min, while the U-Net DR and Swin UNETR take about 10 and 11 min, respectively. In addition, visualizing the segmentation results in 3D Slicer adds an extra 2 min to the total time.

It is important to note that all the methods tested are highly competitive with manual segmentation, which can take up to 2–3 h depending on the complexity of the image and the level of detail required. The proposed methods, therefore, offer a significant reduction in processing time, making them a valuable alternative for clinicians, particularly when dealing with large datasets or needing to process a high volume of cases.

The computations were carried out on a workstation with an Intel Core i5-8500T CPU and 32GB of RAM. In a clinical setting, where specific hardware such as GPUs is often available, these methods could be further optimized and scaled, allowing for faster processing times and more efficient handling of larger datasets.

### Lesion set

**Fig. 8:**
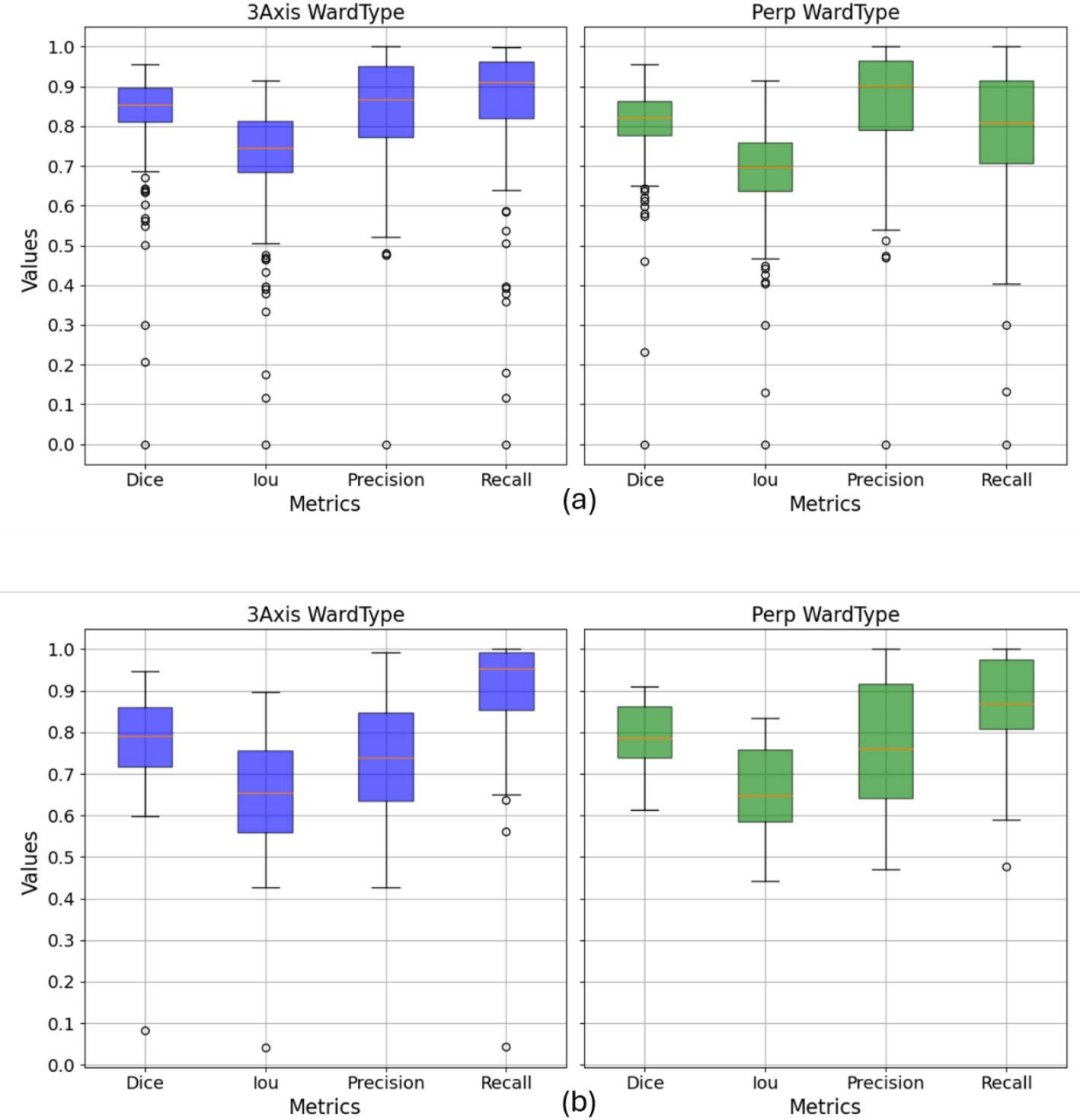
Radial plot displaying the mean parameter values, Dice, IoU, Precision and Recall, obtained by the clustering segmentation algorithms in three views (axial, sagittal, and coronal), referred to as 3Axis, and the segmentation algorithm in the perpendicular view, referred to as Perp in the lesion set. (**a**) Mean values obtained in the whole coronary tree. (**b**) Mean values obtained in a cube of 8 mm centered in the lesion

In the evaluation of the test set, both models demonstrate highly similar performance, as depicted in Fig. 8a. A notable observation is the decrease in recall for the 3Axis method, exceeding 10%, while maintaining precision. Conversely, in the case of the Perp method, recall increases while precision decreases, with both metrics experiencing an 8% shift. Additionally, in Fig. 8b, we observe the performance within a cube of size 32 pixels (8 mm), cantered in the lesion. Once again, recall values surpass 90% for both methods, while precision decreases by more than 10%, resulting in oversegmented lesions.

To evaluate the robustness of the method, a comparison using datasets of 11 and 22 patients is included in Section S5 of the Supplementary Material. In addition, Hausdorff Distance and Mean Surface Distance (MSD) metrics can be found in Supplementary Material, Section S6.

**Fig. 9:**
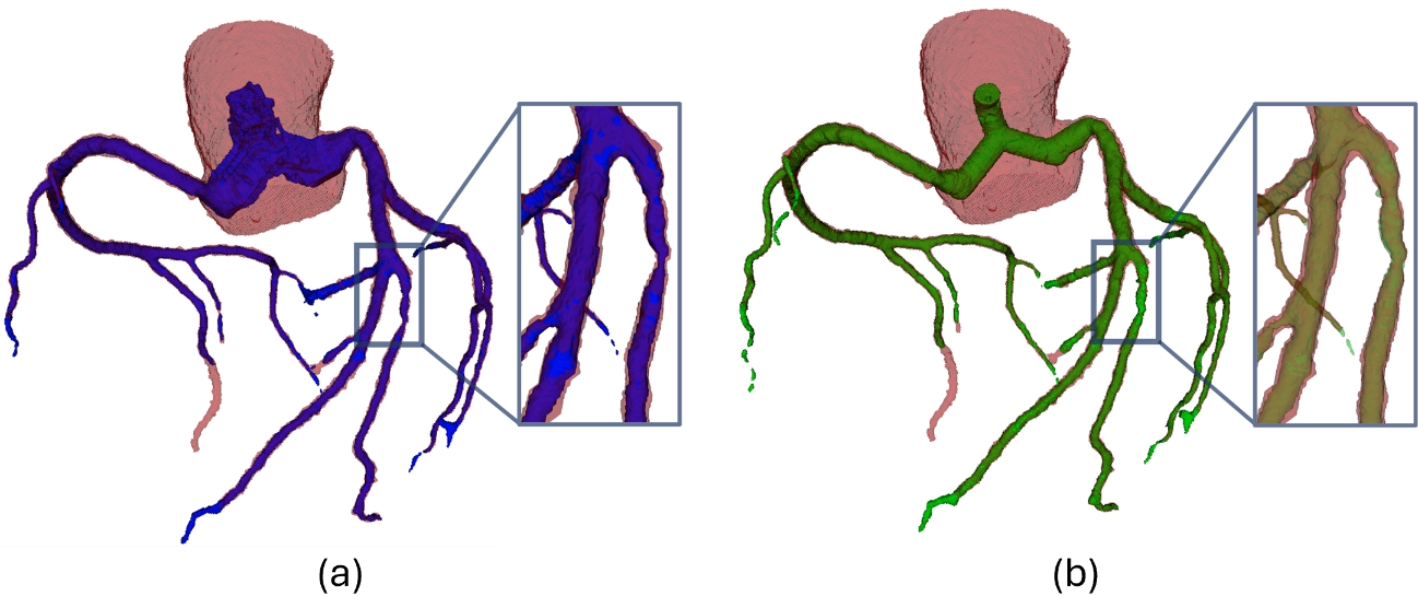
3D geometries in a patient with a lesion. The lesion is located at a bifurcation, where poor segmentation can alter the flow distribution in different branches and affect the circulation in the coronary tree. Ground truth is depicted in red, with the lesion region highlighted in the detailed plane. (**a**) Segmentation performed by the 3Axis clustering algorithm is shown in blue. (**b**) Segmentation carried out by the Perp clustering algorithm is represented in green

**Fig. 10:**
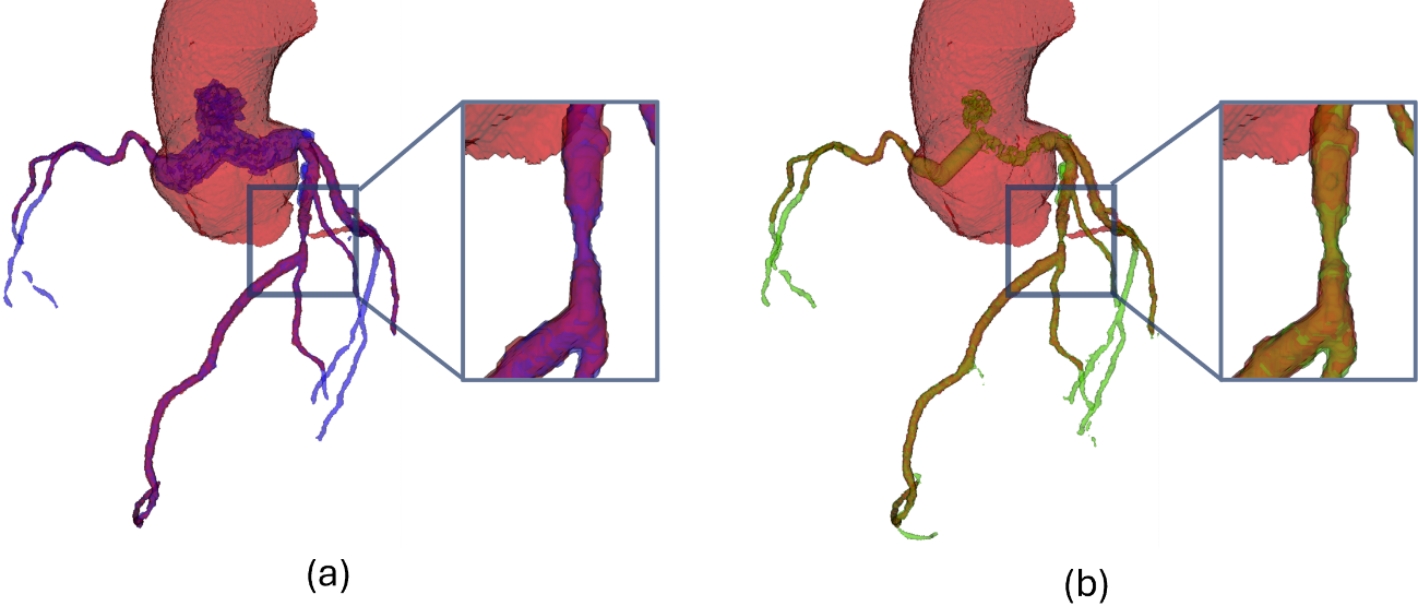
3D geometries in a patient with a lesion. The lesion is a pronounced stenosis in a straight segment of the vessel. Poor segmentation can lead to high fluctuations in clinically relevant diagnostic parameters such as FFR. Ground truth is depicted in red, with the lesion region highlighted in the detailed plane. (**a**) Segmentation performed by the 3Axis clustering algorithm is shown in blue. (**b**) Segmentation carried out by the Perp clustering algorithm is represented in green

In addition to metrics, obtaining a visual representation of lesions is crucial. Figures 9 and 10 showcase segmentation examples for patients with lesions. In both cases, both algorithms successfully identify vessel narrowing (stenosis). However, in Fig. 9, both algorithms underestimate the vessel, resulting in narrower segmentations, although Perp overestimates the lesion width. Conversely, in Fig. 10, both algorithms slightly overestimate the lesion width. Additionally, the segmentation reveals fat adhered to the vessel in the upper region of the lesion, which both methods mistakenly identify as vessel due to its brightness value in the medical image.

## Discussion

In this work, we propose and compare two coronary artery segmentation methods based on the Ward clustering algorithm. The first method uses images from axial, sagittal, and coronal planes (3Axis), while the second method uses cross-sectional images (Perp). Both methods were tested on two datasets: the first consisting of 10 patients without lesions (test set) and the second consisting of 22 patients with 30 clinically diagnosed lesions (lesion set). The highest Dice score was obtained by the 3Axis method, achieving values of 0.88 for the test set and 0.83 for the lesion set (compared to 0.81 and 0.82 for the Perp method, respectively).

Our results indicate that the 3Axis method outperforms the Perp method in both datasets. This is consistent with the findings of [16, 26]. Furthermore, the 3Axis method’s performance on the same test dataset surpasses that of 2.5D neural networks such as EfficientNet, VGG, ResNet, and U-Net + + in terms of Dice coefficient. Among all 3D network approaches, only the transformer-based Swin UNETR managed to surpass the 3Axis Ward method, achieving a slightly higher Dice score by 0.1. While the 3D U-Net utilizes spatial information across slices to enhance segmentation, the 2.5D Ward method still delivers highly competitive results. This highlights the robustness and effectiveness of clustering techniques in accurately segmenting vascular lesions, even without the complexity of 3D neural network architectures.

This presents a significant computational and data advantage, as our method does not require special resources or a training dataset.

Additionally, our segmentation algorithm, particularly in handling the kissing vessel artifact, does not need information from previous slices, like [14], as the segmentation is performed independently point by point. This enhances the method’s robustness and accuracy.

Interestingly [27], emphasized the importance of multi-objective clustering for coronary artery segmentation, similar to our approach. However, their reliance on a toroidal model for vessel tracking resulted in a lower Dice coefficient, highlighting the effectiveness of our multi-plane imaging technique.

Our clustering-based segmentation method also achieves performance directly comparable to some of the state-of-the-art deep learning approaches implemented for coronary artery segmentation [13].

Huang et al. [47], for instance, employs a deep learning approach with vessel-centered input images and an encoder-decoder architecture featuring a 3D encoder and a 2D decoder. Trained on a small dataset of 29 patients (5 for testing), this method achieves a Dice score of 0.86, slightly lower than the results of our clustering algorithms. Notably, our method remains competitive despite requiring less computational complexity and training data.

Gu et al. [48], by contrast, implements a V-Net architecture using full-image volumes (128 × 128 × 60) and a larger training dataset (70 patients, 20 for testing). This method achieves a higher Dice score of 0.91, but it lacks the focus on vessel-specific segmentation that our approach provides. Moreover, our method excels in lesion segmentation, maintaining clinical relevance and accuracy in these challenging regions.

One of the key advantages of our approach is the detailed and clinically validated segmentation it achieves, made possible using accurate manual segmentation as ground truth.

In terms of dataset size, we acknowledge the limitation; however, we evaluated a total of 30 clinically diagnosed lesions based on highly meticulous manual segmentations. This highlights the high precision achieved by the algorithms. The identification of lesion regions poses a significant challenge, as it must be performed automatically. When predicting for a new patient, we do not have prior knowledge of the location or extent of lesions. Furthermore, it is likely that some lesions are not identified even by clinicians, adding complexity to the task. Despite these challenges, we believe that as a first iteration, the algorithm’s results are very good; while the segmentation may not be as precise in some cases, the lesions are still successfully recognized. As future work, we aim to enhance the detection and segmentation of these challenging regions automatically.

The inclusion of additional vessels in the segmentation process depends on whether a centerline is already available for those vessels, which is independent of the clustering algorithm itself. In this study, we focused primarily on accurately reproducing the main vessels, with particular attention given to areas with lesions. However, smaller-calibre or highly distal vessels were also considered when centerlines were present. The calibre of the vessel plays a significant role in clinical decision-making. Accurate vessel calibre measurements enable the resulting geometries to be integrated into computational fluid dynamics (CFD) simulations to derive clinically relevant parameters, such as fractional flow reserve (FFR). Moreover, precise measurements of vessel calibre are critical for determining parameters like the degree of stenosis in a lesion, which directly inform intervention decisions.

Although the scarcity of data has limited a comprehensive study with other types of images or scanners, the promising results suggest the generality of the method. The precision and complete segmentation of the coronary tree achieved in this study present a significant advancement over previous approaches. Incorporating a tracking algorithm to make the method fully automatic and allowing more flexibility in selecting the number of clusters or increasing the threshold decision algorithm’s complexity could further refine the segmentation process and enhance overall accuracy.

Overall, our study demonstrates the robustness of the 3Axis method in various clinical scenarios, including cases without lesions and those with clinically diagnosed lesions. Future research could focus on integrating additional imaging modalities and refining the algorithm to further improve segmentation accuracy, particularly in vessels with low contrast or calcified lesions.

## Conclusions

In conclusion, we introduce a novel clustering method for segmentation that eliminates the need for training, offering a streamlined approach to image analysis. Furthermore, our method provides valuable insights into the location and connections of clusters through a complex network representation. We validated our approach using coronary artery data, a challenging dataset due to the complex geometry of the vessels and the inherent motion artifacts in cardiac images. While our primary focus was on artery segmentation, we are confident that our algorithm has broader applicability and can be successfully applied to other anatomical structures. This study lays the groundwork for further exploration and implementation of our clustering method in diverse medical imaging contexts, promising advancements in automated segmentation techniques and facilitating more accurate diagnostic assessments.

## Electronic supplementary material

Below is the link to the electronic supplementary material.

Supplementary Material 1

## Data Availability

No datasets were generated or analysed during the current study.

## Abbreviations

CT: Computed Tomography
CTCA: Computed Tomography Coronary Angiography
AI: Artificial Intelligence
SMF: Symmetrical Radiation Filter
CRF: Case Report Form
TP: True Positives
FP: False Positives
TN: True Negatives
FN: False Negatives
IoU: Intersection Over Union
