# Supplementary material for: Unsupervised clustering based coronary artery segmentation
Source: BioData Min. 2025 Mar 7;18:21. doi: 10.1186/s13040-025-00435-y (PMC11887207; doi:10.1186/s13040-025-00435-y)
Supplement: Supplementary file 1 — Supplementary Material 1 [file 13040_2025_435_MOESM1_ESM.docx]

Supplementary Material. Unsupervised clustering based coronary artery segmentation

Belén Serrano-Antón^a,b,c^, Manuel Insúa Villa^a^, Santiago Pendón Minguillón^a^, Santiago Paramés-Estévez^a,b,c^, Alberto Otero-Cacho^a,b,c,∗^, Diego López-Otero^d,e^, Brais Díaz-Fernández^d,e^, María Bastos-Fernández^d,e^, José R. González-Juanatey^d,e,f^, Alberto P.Muñuzuri^b,c^

1. *FlowReserve Labs S.L., Santiago de Compostela, 15782, Galicia, Spain*
2. *CITMAga, Santiago de Compostela, 15782, Galicia, Spain*
3. *Group of Nonlinear Physics. University of Santiago de Compostela, Santiago de Compostela, 15782, Galicia, Spain*
4. *Cardiology and Intensive Cardiac Care Department, University Hospital of Santiago de Compostela, Santiago de Compostela, 15706, Galicia, Spain*
5. *Centro de Investigación Biomédica en Red de Enfermedades Cardiovasculares (CIBERCV), Madrid, 28029, Madrid, Spain*
6. *Instituto de Investigación Sanitaria de Santiago de Compostela (IDIS), Santiago de Compostela, 15706, Galicia, Spain*

*Corresponding author

Email address: [alberto.otero@flowreserve.es](mailto:alberto.otero@flowreserve.es) (Alberto Otero-Cacho)

1. **Ethics Statement**

The development of the project was carried out respecting the Declaration of Helsinki of the World Medical Association 1964 and ratifications of the following assemblies (Tokyo 75, Venice 83, Hong Kong 89, Somerset West 96, Scotland 00, Seoul 08 and Fortaleza 13) on ethical principles for medical research on human beings, RD 1090/2015, of December 24, on clinical trials, specifically the provisions of article 38 on good clinical practices, and the Convention on human rights and biomedicine), made in Oviedo on April 4, 1997 and successive updates. The researchers participating in this study agree that all clinical data collected from the study subjects will be separated from personal identification data ensuring the anonymity of the patient; respecting the Personal Data Protection Law (Organic Law 15/1999, of December 13), RD 1720/2007 of December 21, which approves the Regulations for the development of Organic Law 15/1999, Law 41/2002, of November 14 (basic regulation of patient autonomy and rights and obligations in terms of information and clinical documentation), as well as Law 3/2001, of May 28, (regulator of informed consent and the clinical history of patients), Law 3/2005, of March 7, modifying Law 3/2001 and Decree 29/2009 of February 5, which regulates access to history electronic clinic. The clinical data of the patients will be collected by the investigator in the Case Report Form (CRF) specific to the study. Each CRF will be encrypted, protecting the identity of the patient. Only the research team and the health authorities, who have a duty to maintain confidentiality, will have access to all the data collected for the study. Only information that cannot be identified may be transmitted to third parties. Once the study is finished, the data will be destroyed. The treatment, communication and transfer of data will be done in accordance with the provisions of the General Data Protection Regulation (Regulation (EU) 2016/679 of the European Parliament and of the Council, of April 27, 2016). The data collected will only be used for the purposes of the research study described in the protocol and kept for the time necessary to achieve the objectives of the study and in accordance with applicable legislation. As this is a retrospective study of medical records and archived samples that does not deviate from routine clinical practice, the Ethics Committee consider that patient informed consent and fully anonymization of the data before being access are sufficient requirements to carry out the study.

1. **Manual segmentation details**

Manual segmentation was conducted on contrast-enhanced cardiac images obtained via Coronary Computed Tomography Angiography (CCTA). The focus was on identifying and delineating the main coronary arteries—Left Coronary Artery (LCA), Circumflex Artery (Cx), and Right Coronary Artery (RCA)— and secondary bifurcations to establish precise vessel boundaries.

**Step 1: Pre-Segmentation Calibration**

The segmentation process began with calibrating the brightness window in the analysis software. The average brightness of blood in the region between the aorta and the ostium, where the coronary arteries originate, was measured in Hounsfield Units (HU). This value was used to optimize the visibility of the coronary arteries by adjusting the range of densities highlighted in the images.

**Step 2: Vessel Segmentation**

Once the brightness window was calibrated, the expert segmented the coronary arteries by tracing their boundaries:

- The minimum brightness at the vessel edges was used to accurately define the vessel’s caliber.
- The maximum brightness was determined using the proximal aorta as a reference for blood contrast enhancement.

Calcified atherosclerotic plaques, which exhibit significantly higher HU values (typically 600–1200 HU), were carefully distinguished from blood (200–600 HU) to ensure accurate delineation, particularly in vessels with calcifications or irregular structures.

**Step 3: Adjustments for Patient-Specific Variability**

Brightness and density thresholds were not fixed but tailored for each patient to account for individual variations in tissue attenuation and blood density. This approach ensured accurate segmentation aligned with the anatomical and pathological characteristics unique to each patient.

**Step 4: Software Utilization**

The segmentation was performed using 3D Slicer, an open-source medical imaging tool. DICOM-format images were loaded into the software, allowing precise interaction with three-dimensional image volumes. Experts meticulously delineated the vessels to ensure high reproducibility of the ground truth data.

Observer Variability Analysis

To evaluate intra-observer and inter-observer variability, we conducted a study measuring pressures at corresponding geometric points within the 3D coronary geometry using computational fluid dynamics (CFD) simulations.

- Intra-Observer Variability:
  - Intraclass Correlation Coefficient (ICC): 0.867
  - ICC 95% Confidence Interval [0.51–1]
  - Pearson Correlation Coefficient: 0.93
  - Number of samples (N): 8
- Inter-Observer Variability:
  - Intraclass Correlation Coefficient (ICC): 0.669
  - ICC 95% Confidence Interval [0.48–0.859]
  - Pearson Correlation Coefficient: 0.817
  - Number of samples (N): 71

This evaluation demonstrates the reliability of the manual segmentation process. The use of CFD to analyze pressure differences at identical geometric points reinforces the robustness and clinical relevance of the segmentation protocol.

1. **Justification for the Use of the Ward Clustering Algorithm**

In this study, we aimed to select a clustering algorithm that is robust and generalizable. Specifically, the chosen algorithm needed to perform consistently across different patients, variations in brightness distributions (measured in Hounsfield Units), and diverse anatomical views (axial, sagittal, and coronal).

We evaluated several clustering algorithms from *sklearn.cluster* under the same configuration:

1. KMeans with 7 clusters.
2. DBScan with parameters $eps=3.5$ and $min\_samples=5$.
3. Agglomerative Ward clustering with 7 clusters (denoted as Ward).
4. Agglomerative Ward clustering with grid connection using *grid_to_graph* from *sklearn.feature_extraction.image* (denoted as Ward*, the algorithm used in this study).
5. Spectral clustering with $affinity=nearest\_neighbors$and $random\_state=0$.

The primary objective was to identify an algorithm capable of delineating the vessel's concentric edges (from the interior to the exterior), consistent with the radial decay in brightness.

Figure S 1 to Figure S 3 illustrate the original image and the labels generated by these clustering algorithms applied to different vessels of the same patient.

- Figure S 1 presents a sagittal view of a vessel. In this example, DBScan produces highly irregular clustering patterns at the vessel boundary, unlike the other algorithms. However, only Ward* achieves uniform clusters across the entire vessel, including the boundary.
- Figure S 2 displays an axial view, nearly perpendicular to the vessel. While all algorithms (except DBScan) produce circular and homogeneous clusters within the vessel lumen, only Ward* maintains this uniformity at the boundary.
- Figure S 3 provides a contrasting example where DBScan generates homogeneous and well-defined clusters at the boundary. However, this result highlights DBScan's lack of generalizability, as it fails to maintain consistency across examples from the same patient.

Based on these results, Ward* was selected for this study due to its ability to produce homogeneous and compact clusters consistently across different views, vessels, and conditions. This ensures reliability and reproducibility in segmenting the vessel edges, critical for the objectives of this study.


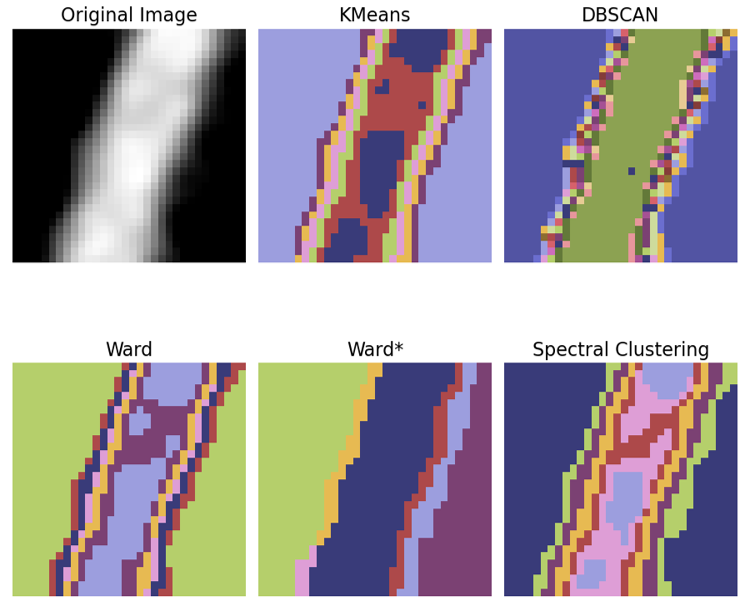


Figure S 1. Results of different clustering algorithms—KMeans, DBSCAN, Ward, Ward* (our method), and Spectral Clustering—are displayed, showcasing their respective segmentations of the longitudinal section of a vessel showing its axis in a sagittal plane. The vessel exhibits a smooth and regular border, with no visible irregularities.


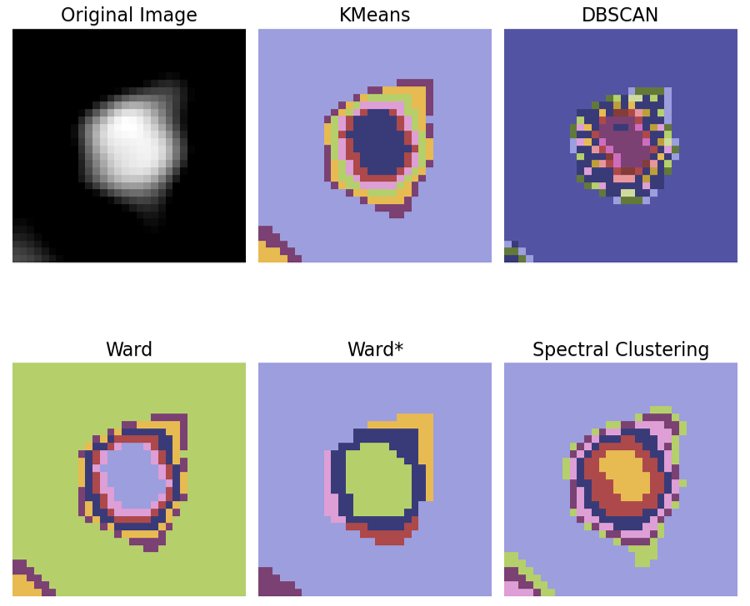


Figure S 2. Results of different clustering algorithms—KMeans, DBSCAN, Ward, Ward* (our method), and Spectral Clustering—are displayed, showcasing their respective segmentations of the axial view of the vessel, nearly perpendicular to the centerline, showing a circular shape characteristic of its structure.


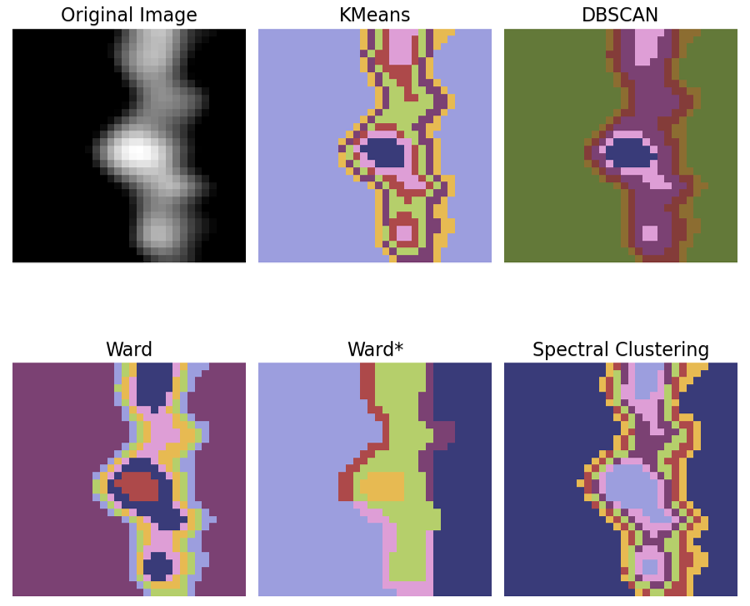


Figure S 3. Results of different clustering algorithms—KMeans, DBSCAN, Ward, Ward* (our method), and Spectral Clustering—are displayed, showcasing their respective segmentations of the longitudinal section of the vessel in the coronal plane, displaying notable lesions and irregularities along the vessel's border.

1. **Examples of Ward’s method clusters and ground truth segmentation**


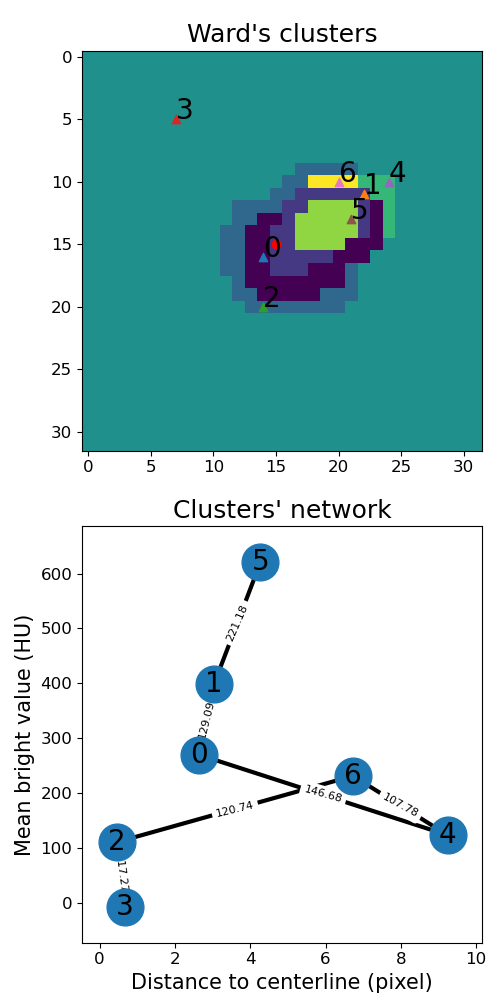


Figure S 4. Visualization of Ward’s clustering results and the graph structure generated for the vessel depicted in Figure 7. The clusters are color-coded, representing distinct intensity regions, including the background, vessel interior, and calcium deposits. The graph structure establishes an ordered hierarchy from the vessel’s interior to its edge, enabling an intuitive interpretation of the segmentation. This arrangement facilitates the identification of pathological features and provides a comprehensive view of the vessel structure.


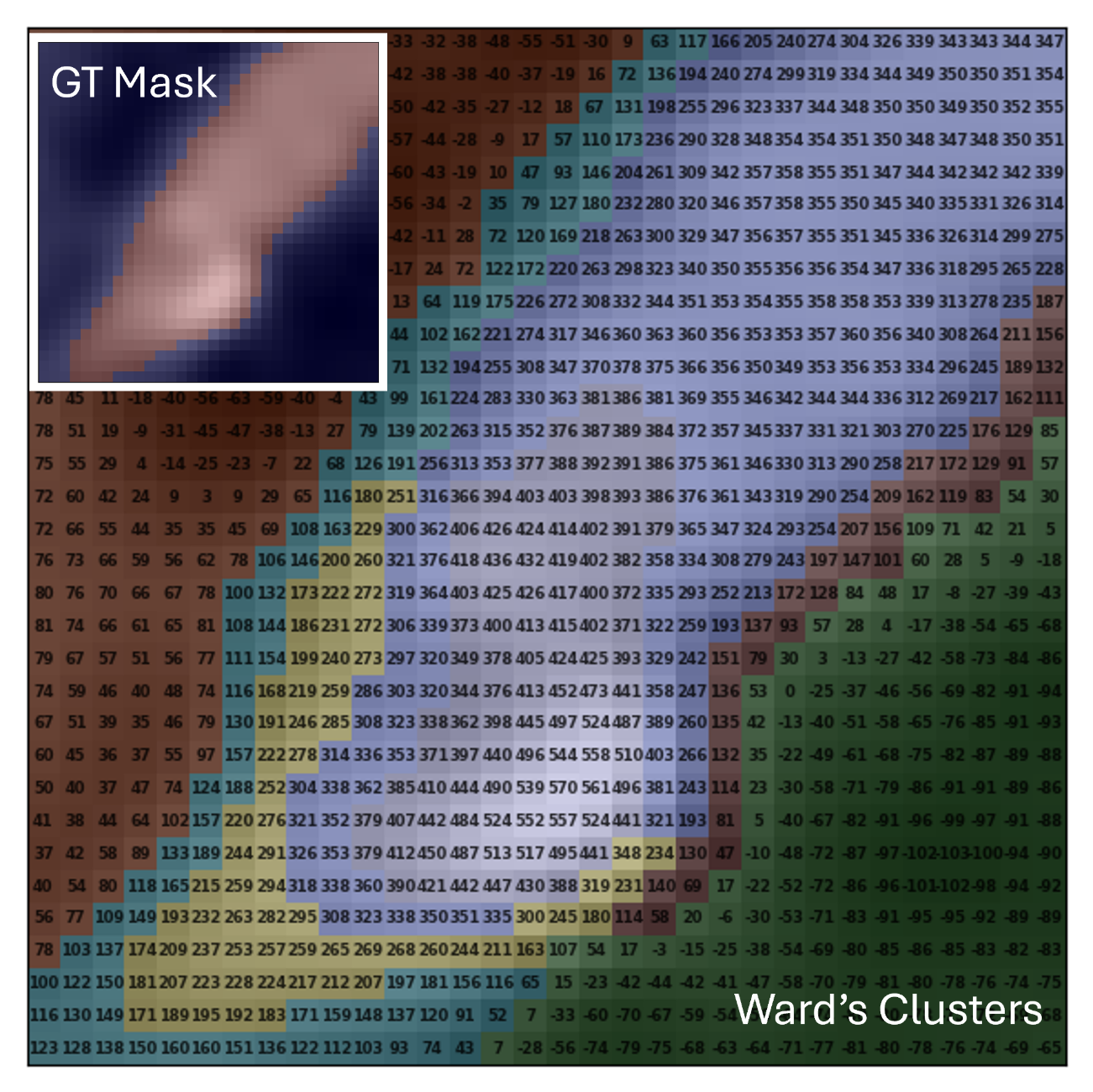


Figure S 5. Results of the clusters obtained using the Ward algorithm after applying background removal and the corresponding ground truth mask, for a vessel with no lesion in coronal view. The brightness value of each pixel in the original image is displayed as its corresponding HU value directly on the pixel


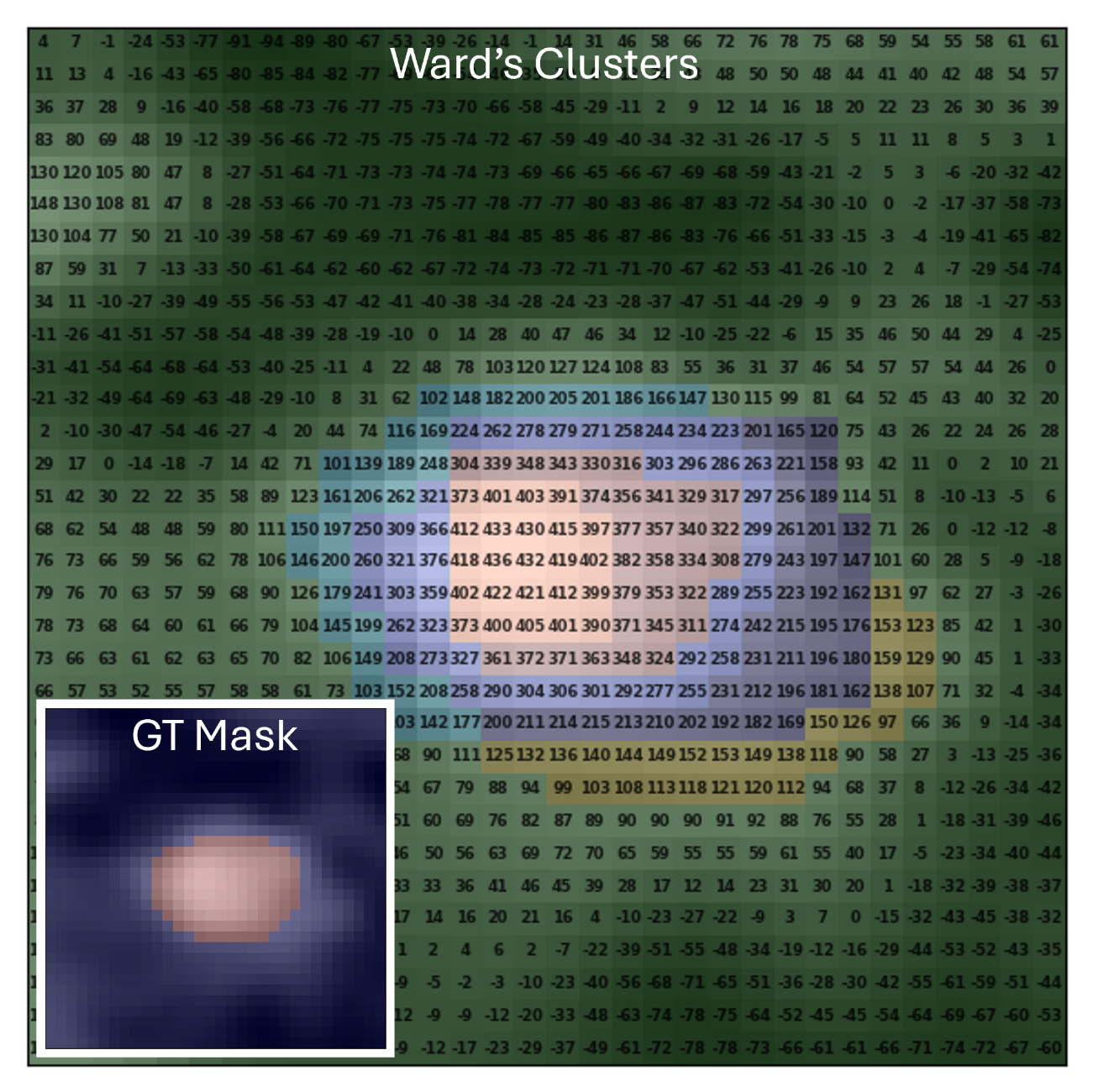


Figure S 6. Results of the clusters obtained using the Ward algorithm after applying background removal and the corresponding ground truth mask, for a vessel with no lesion in cross-sectional view. The brightness value of each pixel in the original image is displayed as its corresponding HU value directly on the pixel

1. **Comparison 11 – 22 lesion patients**

In Figure S 6, the boxplots showing the results of various metrics (Dice, IoU, Precision, Recall, and Accuracy) for 11 patients from the lesion set and 22 patients from the lesion set are presented. It can be observed that there are more outliers in the 22-patient group, likely due to incomplete segmentation of certain branches, as lower recall values are also observed. Despite this, both 3Axis and Perp demonstrate robustness when including additional patients. Furthermore, 3Axis shows slightly higher median values, indicating a potential advantage in performance.


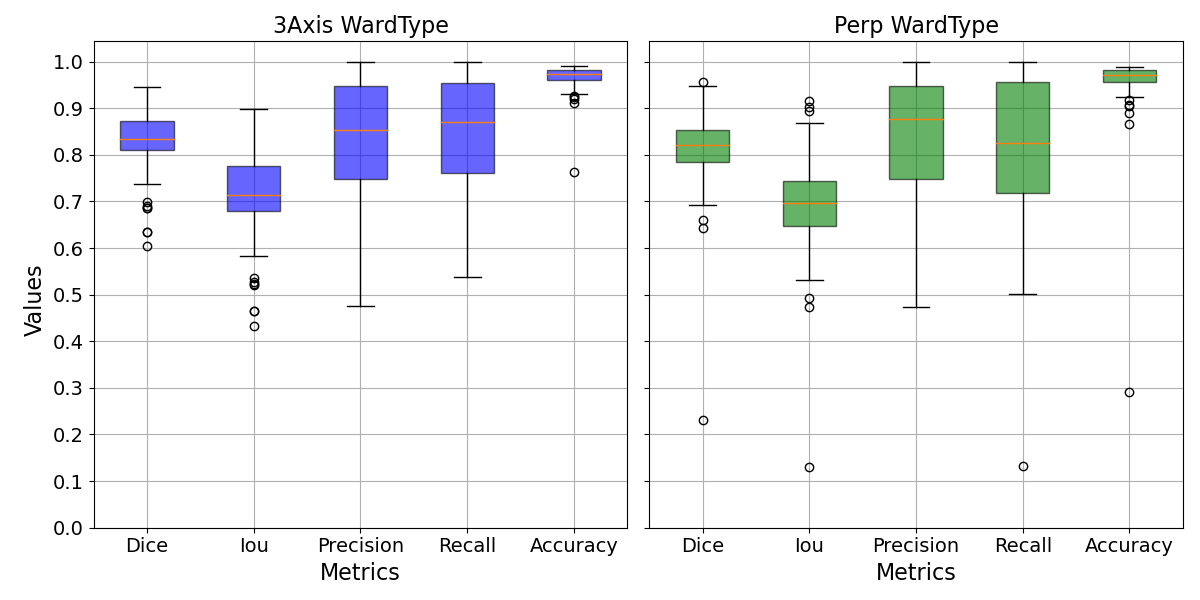

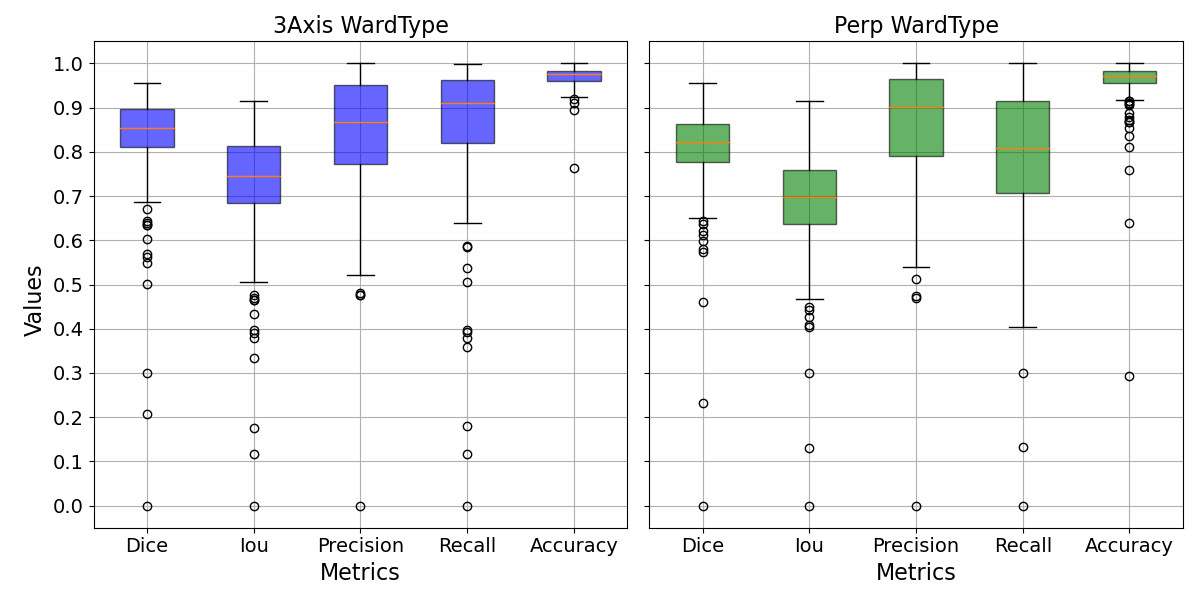


(a)

(b)

Figure S 7. Performance metrics Dice, IoU, Precision, Recall, and Accuracy for 3Axis and Perp. (a) with 11 patients with lesions. (b) with 22 patients with lesions.

1. **Hausdorff Distance and Mean Surface Distance**

We present the Hausdorff Distance and Mean Surface Distance (MSD) metrics for the test set. In Figure S 7(a), we observe the values for the clustering-based algorithms 3Axis and Perp. Some outliers are visible, likely due to areas where the vessel was not segmented (areas with low recall). On average, the values are 4.796 (std: 3.373) for 3Axis and 4.747 (std: 3.852) for Perp, with both values being very similar.

Turning our attention to the 2.5D neural networks (Figure S 7(b)), fewer outliers are observed, indicating more consistent results. However, the mean values for the Hausdorff distance range from 9.56 to 11.553, with a standard deviation around 4. These values are nearly double those of the clustering-based algorithms.

The 3D networks, specifically 3D U-Net and Swin UNETR, exhibit lower values of Hausdorff Distance compared to the 2.5D networks. For example, the Hausdorff Distance for 3D U-Net is mean: 5.8607 with a standard deviation of 4.0450, while Swin UNETR shows mean: 6.0710and a standard deviation of 4.298. On the other hand, the Ward method, as a 2.5D approach, achieves lower Hausdorff Distance values, highlighting its ability to generate more precise segmentations in comparison. Notably, U-NetDR performs the worst in terms of Hausdorff Distance, with a mean of 18.4929 and standard deviation of 3.5859, indicating a significant gap between its segmentation results and the ground truth.

Regarding the MSD, very similar values are observed across all models. The mean and standard deviation (std) values can be seen in Table S 1.


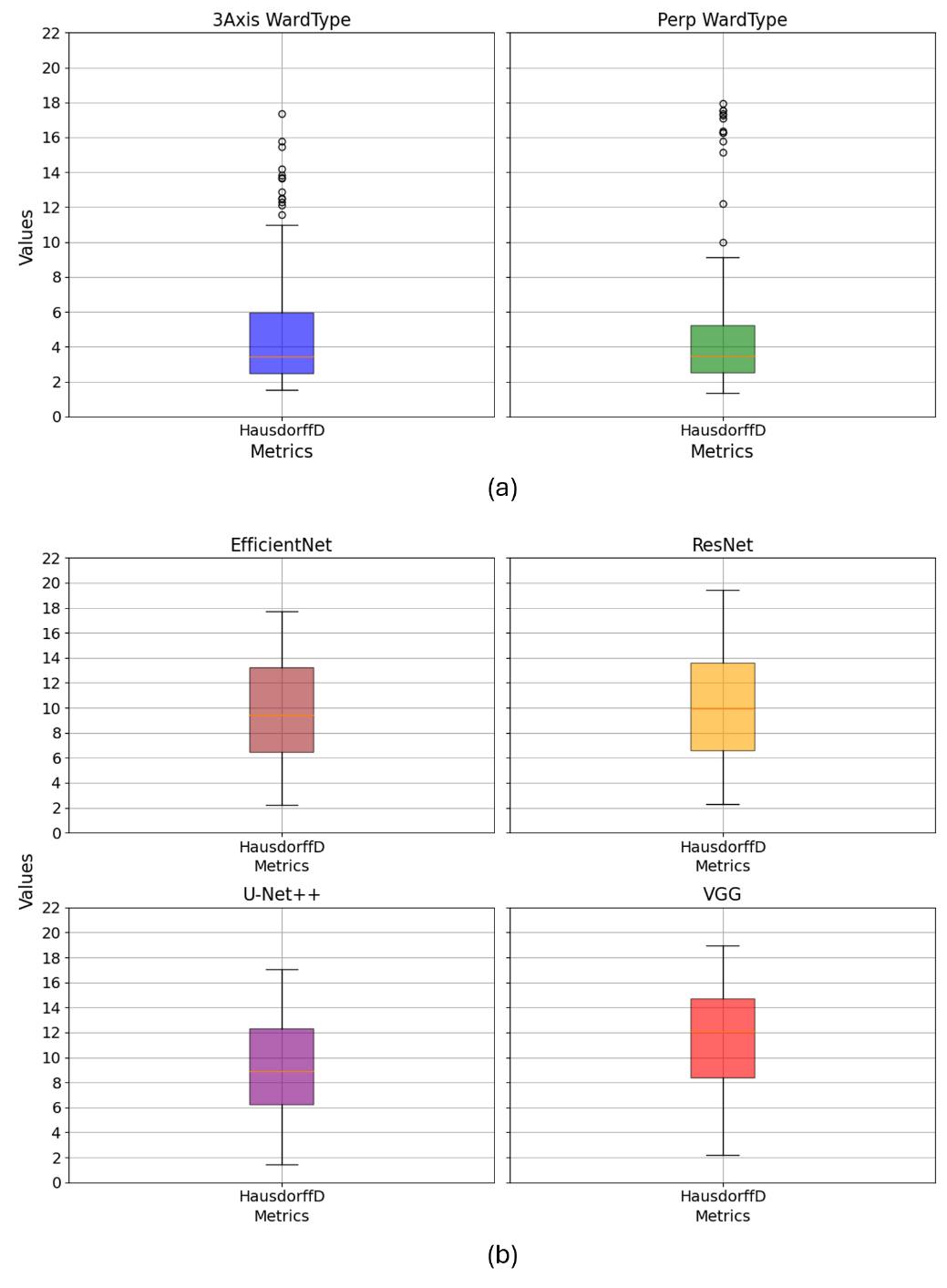


Figure S 8. Box plots of the Hausdorff Distance metric between the ground truth and predictions for the 10 test set patients. (a) Comparison of 3Axis and Perp ward methods. (b) Performance of neural network models: EfficientNet, ResNet, U-Net++, and VGG.


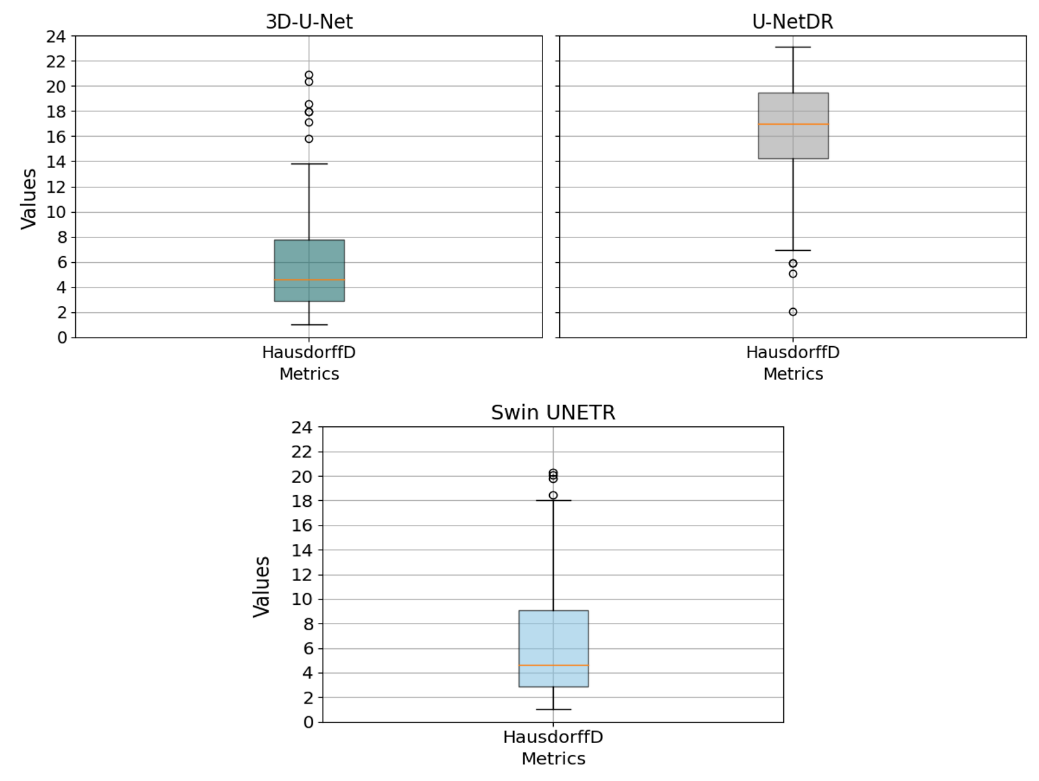


Figure S 9. Box plots of the Hausdorff Distance metric between the ground truth and predictions for the 10 test set patients by the 3D models U-Net, U-Net DR and Swin UNETR.

| Model | Mean MSD | Std MSD |
| --- | --- | --- |
| 3Axis-Ward | 0.1276 | 0.8546 |
| Perp-Ward | 0.1576 | 0.8730 |
| EfficientNet | 0.1390 | 0.8764 |
| ResNet | 0.1380 | 0.8772 |
| U-Net++ | 0.1342 | 0.8724 |
| VGG | 0.1474 | 0.8769 |
| 3D U-Net | 0.0521 | 0.4108 |
| 3D U-Net DR | 0.2589 | 0.2776 |
| 3D Swin UNETR | 0.1052 | 0.8223 |

Table S 1. Mean values and standard deviations (std) of the Mean Surface Distance (MSD) for the 3Axis and Perp methods, as well as the neural networks EfficientNet, ResNet, U-Net++, and VGG, 3D U-Net and 3D U-Net DR, evaluated on the 10 test patients.

In the lesion patients, consistent with the other metrics, the Hausdorff Distance increases slightly. The mean values reach 5.278 (std: 3.501) for 3Axis and 5.615 (std: 3.553) for Perp (see Figure S 9(a)). Additionally, in the lesion region (see Figure S 9(b)), there is an increase in the mean values by up to 3 points.

However, a slight reduction in the MSD is observed (see Table S 2), which may indicate that the edges of the segmentation are closer to the ground truth, despite the lower intersection volume (as measured by metrics such as Dice).


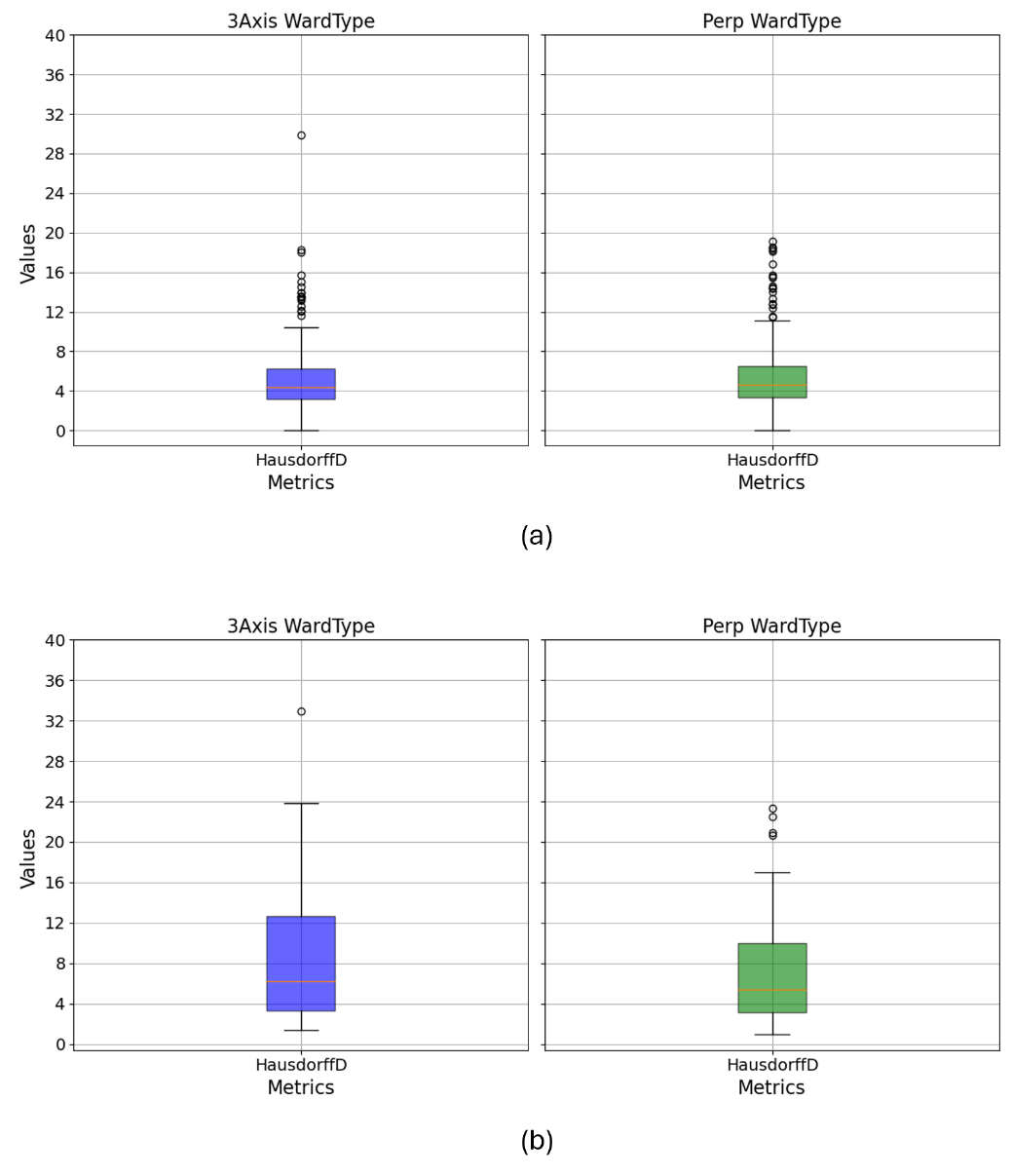


Figure S 10. Box plots of the Hausdorff Distance metric between the ground truth and predictions for the lesion set using the 3Axis and Perp methods. (a) Comparison for the 22 patients in the lesion set. (b) Comparison for the 30 lesions (in a cube of 8 mm centered in the lesion) in the lesion set.

| Model | Mean MSD | Std MSD |
| --- | --- | --- |
| 3Axis-Ward | 0.0631 | 0.6334 |
| Perp-Ward | 0.0784 | 0.6514 |
| 3Axis-Ward in Lesion | 0.0284 | 0.0218 |
| Perp-Ward in Lesion | 0.0251 | 0.0172 |

Table S 2. Mean values and standard deviations (std) of the Mean Surface Distance (MSD) for the 3Axis and Perp methods, evaluated on the 22 patients in the lesion set and the 30 lesions in the lesion set (in a cube of 8 mm centered in the lesion).
